# The structure of the symptoms of major depression: Factor analysis of a lifetime worst episode of depressive symptoms in a large general population sample

**DOI:** 10.1016/j.jad.2022.03.064

**Published:** 2022-04-01

**Authors:** Hanna M. van Loo, Steven H. Aggen, Kenneth S. Kendler

**Affiliations:** a University of Groningen, University Medical Center Groningen, Department of Psychiatry, Groningen, the Netherlands; b Virginia Institute for Psychiatric and Behavioral Genetics, Department of Psychiatry, Virginia Commonwealth University, Richmond, VA, USA

**Keywords:** Depressive symptoms, General population, Factor analysis, Symptom dimensions

## Abstract

**Background:**

A range of depressive symptoms may occur during an episode of major depression (MD). Do these symptoms describe a single disorder liability or different symptom dimensions? This study investigates the structure and clinical relevance of an expanded set of depressive symptoms in a large general population sample.

**Methods:**

We studied 43,431 subjects from the Dutch Lifelines Cohort Study who participated in an online survey assessing the 9 symptom criteria of MD (DSM-IV-TR) and additional depressive symptoms during their worst lifetime episode of depressive symptoms lasting two weeks or more. Exploratory factor analyses were performed on expanded sets of 9, 14, and 24 depressive symptoms. The clinical relevance of the identified symptom dimensions was analyzed in confirmatory factor analyses including ten external validators.

**Results:**

A single dimension adequately accounted for the covariation among the 9 DSM-criteria, but multiple dimensions were needed to describe the 14 and 24 depressive symptoms. Five dimensions described the structure underlying the 24 depressive symptoms. Three cognitive affective symptom dimensions were mainly associated with risk factors for MD. Two somatic dimensions –appetite/weight problems and sleep problems—were mainly associated with BMI and age, respectively.

**Limitations:**

Respondents of our online survey tended to be more often female, older, and more highly educated than non-respondents.

**Conclusions:**

Different symptom dimensions described the structure of depressive symptoms during a lifetime worst episode in a general population sample. These symptom dimensions resembled those reported in a large clinical sample of Han-Chinese women with recurrent MD, suggesting robustness of the syndrome of MD.

## 1. Introduction

Episodes of major depression (MD) are defined by a diverse set of symptoms and signs, ranging from depressed mood and feelings of worthlessness to appetite problems and fatigue (American Psychiatric Association, 2013). This set of symptoms characterizes a range of cognitive, emotional and somatic clinical features. In theory, patients can satisfy the diagnostic symptom criteria for MD in at least 227 different ways (Fried and Nesse, 2015; Olbert et al., 2014). The potential heterogeneity in clinical syndromes becomes even larger when taking into account the additional symptoms that often occur during episodes of MD –e.g., feeling tense and nervous, hopelessness, early morning awakening– which are not included in the defining A criteria for MD in the DSM (Fried et al., 2016; Kendler et al., 2018).

Because of the broad range of symptoms that may occur during MD episodes, it has long been questioned whether these symptoms cohere in a unidimensional way. That is, do the symptoms indicate a single common underlying dimension of depression liability or do they reflect multiple underlying dimensions (MacFadyen, 1975; van Loo et al., 2012)? Different dimensions would especially be relevant if they are associated with differences in etiology, prognosis, or treatment response (Wanders et al., 2016). For example, a previous study suggested that different genetic factors underly mood, neurovegetative, and cognitive/psychomotor depressive symptom dimensions (Kendler et al., 2013). Other studies indicated the prognostic value of somatic compared to cognitive symptoms of depression in patients with coronary heart disease (De Jonge et al., 2006; De Miranda Azevedo et al., 2014; Roest et al., 2011).

Although many studies have investigated the dimensionality of depressive symptoms, consistent depressive symptom dimensions have yet to be replicated. Three previous studies in general population samples showed that depressive symptoms in the past weeks or past year were adequately captured by one underlying dimension, but these studies focused mainly on the nine aggregated DSM depressive symptom criteria (Aggen et al., 2005; Van Dam and Earleywine, 2011; Yu et al., 2012). A systematic review of factor analyses in subjects with MD mainly from clinical samples showed at least two dimensions of depressive symptoms, but the nature of these symptom dimensions varied across studies (van Loo et al., 2012). The most consistent finding was that the two core criteria of MD –depressed mood and interest loss–loaded on a common factor. However, the reviewed studies were mostly small (range 96—1049 subjects with MD) and used different assessment and statistical methods. One study using a clinically ascertained sample of more than 6000 Han-Chinese women with recurrent major depressive disorder found that two dimensions accounted for the 9 aggregated DSM-A criteria of depression: a general depressive symptom factor and a guilt/suicidality factor (CONVERGE consortium, 2015; Li et al., 2014). When the disaggregated 14 DSM symptoms plus an additional 13 non-DSM depressive related symptoms were examined, the resulting factorial structure was multidimensional indicating a diverse set of symptoms dimensions such as appetite/weight problems, suicidality/hopelessness, and agitation/anxiety. This study did not investigate whether these symptom dimensions were associated with different etiology, course of illness, or treatment response. In sum, previous studies have provided mixed evidence regarding structures of depressive symptoms during MD episodes, which may be due to differences in the studied samples (clinical or general population), relatively small sample sizes, and the included set of depressive symptoms (van Loo et al., 2012).

Since depressive symptoms may have a different structure and meaning in a clinical population compared to the general population –e. g., sleep problems may reflect the severity of MD in a clinical population, but may be related to aging in a general population (Balsis and Cully, 2008; Ohayon et al., 2004)— it is important to also study the structure and correlates of depressive symptoms in a general population sample. If we study lifetime periods of depressive symptoms in a general population sample, what kind of symptom dimensions do we find, and how are they related to risk factors for MD? The identification of different depressive symptom dimensions may help in our understanding how best to screen for subjects at risk for MD in the general population.

The primary aim of this study is to investigate the dimensional structure of a broad range of depressive symptoms in a Dutch general population sample of 43,431 subjects reporting on their worst lifetime period of depressive symptoms that lasted two weeks or longer. A second aim is to investigate the clinical relevance of the different depressive symptom dimensions by examining their association with well-known risk factors for MD, such as sex, family history of depression/anxiety, and stressful life events.

## 2. Methods

### 2.1. Participants

Data were derived from Lifelines, a multidisciplinary prospective population-based cohort study examining in a unique three-generation design the health and health-related behaviors of 167,729 persons living in the North of the Netherlands (Scholtens et al., 2015). It employs a broad range of investigative procedures to assess the biomedical, sociodemographic, behavioral, physical, and psychological factors that contribute to health and disease in the general population, with a special focus on multimorbidity and complex genetics. For a further description of Lifelines’ study design and characteristics, we refer to previous studies (Klijs et al., 2015; Scholtens et al., 2015). All participants provided written informed consent for participation in Lifelines; the Medical Ethical Committee of the University Medical Center Groningen approved the study protocol. The add-on study “Mood and mental health” (see below) was judged to be exempt from review by the same Medical Ethical Committee because it was not a clinical study with human subjects as meant in the Medical Research Involving Human Subjects Act.

### 2.2. Add-on study “mood and mental health”

In the Summer of 2018, all 124,742 adult Lifelines participants with a working email address were invited to complete an online questionnaire “Mood and mental health”, from an add-on study which was part of the BIObanks Netherlands Internet Collaboration (BIONIC) (Fedko et al., 2020). This online survey assessed the presence of lifetime MD, anxiety disorders, and other mental health related items. The survey consisted of a maximum of 119 questions. Questions were presented in a fixed order with pre-programmed skip patterns for items that were not applicable to reduce the time participants had to spend completing the survey and increase completion rates. A further description of this online survey is provided in the Supplemental Methods. In total, 43,431 (34.8%) of the invited subjects participated in the survey. On average, respondents were more often female, had a higher age, a higher educational attainment and income, and reported fewer episodes of MD or GAD at baseline than non-respondents (Table Supplement 1 (S1)). We did not exclude subjects with other psychiatric disorders such as bipolar disorder, because lifetime diagnoses based on DSM-criteria were not available for most psychiatric disorders in Lifelines.

### 2.3. Depressive symptoms

The DSM symptoms for MD described under criterion A were assessed with the Lifetime Depression Assessment Self-report (LIDAS), largely based on the Composite International Diagnostic Interview (CIDI) (Bot et al., 2017). Participants were asked whether they ever experienced a period lasting at least two weeks with depressed mood and/or anhedonia, almost every day, and if so, which additional symptoms of MD they experienced during this period. Participants were prompted to report these symptoms during the lifetime episode when the feelings of depressed mood or anhedonia were at its worst. If participants reported at least one core symptom of MD (i.e., depressed mood and/or anhedonia), all other depressive symptoms were assessed. If they never experienced a period with any of the core symptoms of MD, the other depressive symptoms were not assessed, in order to focus on additional depressive symptoms that occur in a period when subjects had at least one core symptom of MD. The symptoms appetite, weight, sleep and psychomotor problems –that may either be increased or decreased during a depressive episode– were investigated using separate items so that information was available for all disaggregated items. The validity and feasibility of the Lifetime Depression Assessment Self-report (LIDAS) as a brief instrument for assessing lifetime MD status has been studied in participants of two Dutch cohort studies. Results showed that the LIDAS resulted in adequate levels of both sensitivity and specificity using interviews based on the CIDI as a reference (Bot et al., 2017).

In addition to the DSM-A symptom criteria for MD, a range of other symptoms that can be present during episodes of MD were also assessed, such as loss of libido, feelings of anxiety, hopelessness, and helplessness (Table S2), symptoms that have been reported to occur during depression long before the introduction of the DSM (Kendler, 2017). These other symptoms may play a crucial role in depression symptom networks (Fried et al., 2016; van Loo et al., 2018), and explain part of the clinical heterogeneity. To increase the comparability between the two samples, we chose to assess those additional depressive symptoms that were also assessed in CONVERGE. The items to assess these symptoms were thus derived from the interview used in CONVERGE (CONVERGE consortium, 2015) and were translated from English to Dutch. The quality of the translation was checked using forward and backward translation methods.

### 2.4. External validators

Ten risk variables were selected to investigate the external validity of the different depressive symptom dimensions. These variables were selected based on evidence from previous studies for their demonstrated relatedness to risk for MD (e.g., sex, family history, neuroticism, environmental adversity etc.) (Havinga et al., 2017; Kendler et al., 2006, 2002), or that they may be specifically linked to different symptom dimensions of MD (e.g., age, body mass index (BMI)) (Hegeman et al., 2012; Milaneschi et al., 2017). These ten validators covered different domains:

- Demographics: sex, age at interview, BMI
- Personality: neuroticism
- Familial risk: family history of depression or anxiety in ≥1 first-degree family member
- Environmental adversity: chronic stress, childhood sexual abuse (CSA)
- Psychiatric history: history of MD, history of GAD, history of heavy drinking

For further details about the assessment and definition of these external validators, we refer to the Supplemental Methods.

### 2.5. Statistical analysis

A latent variable common factor modeling approach (Barendse et al., 2015; Flora and Curran, 2004) was used to investigate the structure of three overlapping but progressively expanded sets of both DSM and non-DSM symptoms that can characterize episodes of MD (note that “DSM symptoms” refer to the DSM A-criteria, and “non-DSM symptoms” to symptoms that are not part of the A-criteria). First, exploratory factor analyses (EFA) were carried out that extracted increasing numbers of oblique factors to account for associations among the binary depressive symptoms.

To deal with the missing data on additional depressive symptoms for participants who reported never experiencing either or both of the core MD symptoms for a period of two weeks during their life (~74%, see Table S3 and above), we used a robust full information maximum likelihood (FIML) estimation approach with Monte Carlo integration for the EFA and CFA analyses. Such an approach reduces the impact of selection biases due to the conditional skip-out structure of the interview. The Bayesian-based Monte Carlo integration estimator uses a random resampling procedure to arrive at posterior parameter estimates based on both the observed and missing item data. Because of its Bayesian orientation, this estimation approach is well suited for estimating model parameters when there are substantial amounts of missing data (Givens and Hoeting, 2013). Due to the nature of this random sampling approach, all EFA models were rerun ten times with different random number seeds to take into account that different random starting seeds can produce variability in the global fit indices, although parameter estimates tended to be stable across the different random seeds.

EFAs were performed on three different sets of MD items; 1) nine aggregated DSM-symptoms, 2) fourteen disaggregated DSM-symptoms including separate items for weight (increase and decrease), appetite (increase and decrease), sleep (insomnia and hypersomnia) and psychomotor (retardation and agitation), and 3) the 14 disaggregated plus ten additional non-DSM depressive symptoms, resulting in a total of 24 DSM and non-DSM symptoms. EFA solutions were retained based on recommended statistical fit index cutoffs and structural interpretability. Due to the large sample size, EFA solutions with increasing numbers of factors produced statistically significant improvements in the omnibus fit indices (Table S4) but lacked clinical interpretability, and were dropped from further consideration. These solutions included factors marked by only two items, high inter-factor correlations, or were defined by low factor loadings.

**Fig. 1.**
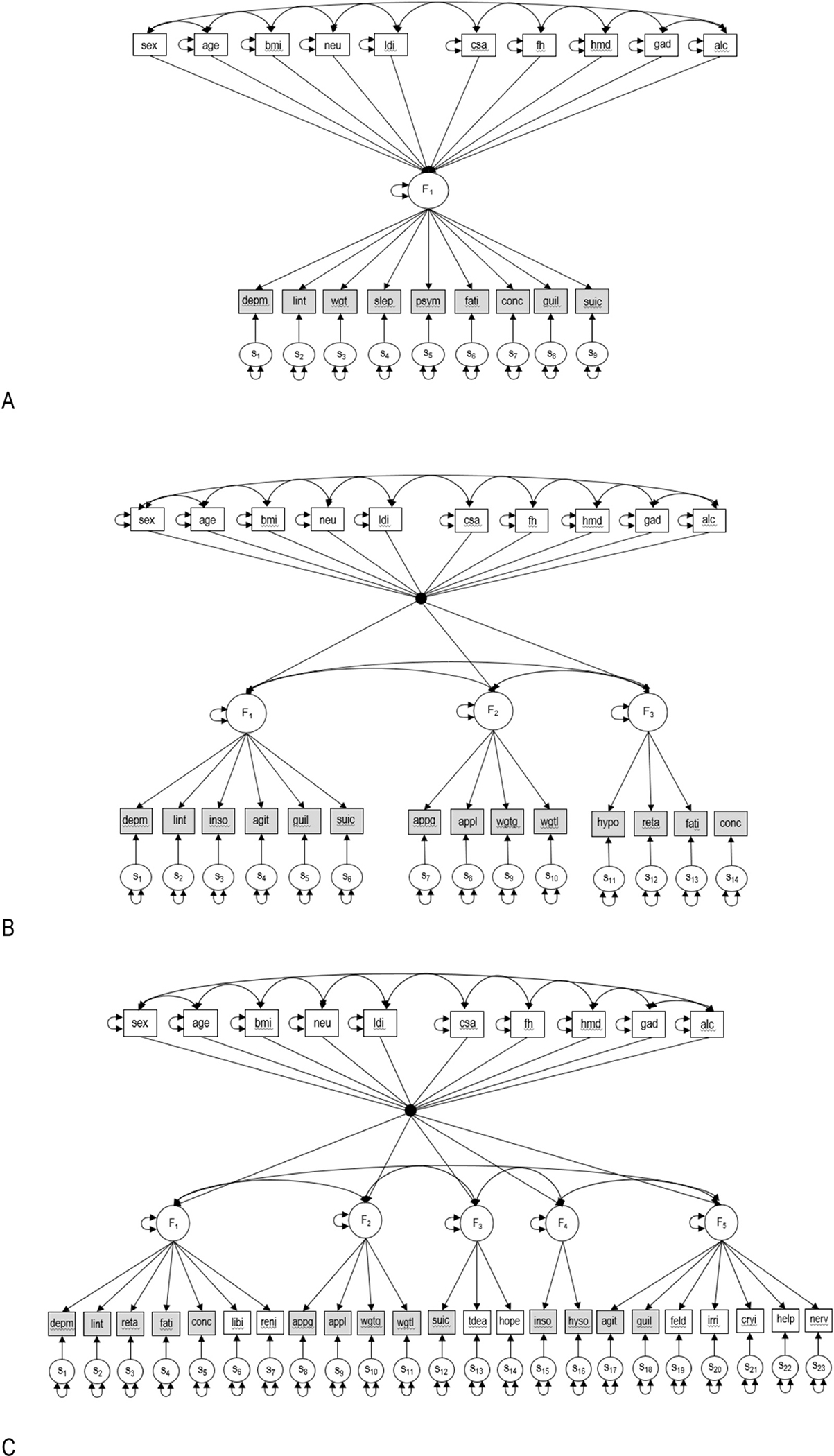
Path diagrams of the correlated external prediction models for the 1 (A), 3 (B), and 5 (C) oblique factor CFA models for the different MD symptom sets. Symptom abbreviations: (A) Depm, depressed mood; lint; loss of interest; wgt, weight problems; slep, sleep problems; psym, psychomotor problems; fati, fatigue; conc, concentration problems; guil, feelings of guilt; suic, suicidal ideation; (B) inso, insomnia; agit, psychomotor agitation; appg, appetite gain; appl, appetite loss; wgtg, weight gain; wgtl, weight loss; hyso, hyposomnia; reta, psychomotor retardation; (C) libi, libido loss; renj, reduced enjoyment; tdea, thoughts of death; hope, hopelessness; feld, different sadness; irri, irritability; cryi, crying; help, helplessness; nerv, nervousness (see also Table S2). External validator abbreviations: bmi, body mass index; neu, neuroticism; ldi, long-term difficulties inventory, csa, childhood sexual abuse; fh, family history of depression and/or anxiety; hmd, history of MD; gad, history of GAD; alc, history of heavy alcohol use. Notes: Square boxes represent observed variables. Circles indicate unobserved variables (factors). Directed arrows (→) with lines emanating from factors and terminating with an arrowhead at a square box are factor loadings. Directed arrows (→) with lines emanating from a square box denoting the external covariates and terminating with an arrowhead at the factors are partial regression coefficients. Shaded item factor indicators (boxes) are binary DSM symptoms and clear indicators are the non-DSM symptoms. Double headed arrows running between different boxes or circles are correlations whereas a double headed arrow attached to a box or circle indicates the variance of that variable. Circles at the bottom of the figure with arrows pointing toward the MD symptom indicators (labeled s_#_) are the specific components of the factor decomposition and represent symptom specific variation not shared with the other symptoms plus random measurement error. The black dot used in the B and C path diagrams is a symbol used to reduce the graphic clutter of having to draw directed arrows from each of the external predictors to each of the common factors.

After identifying statistically supported and substantively interpretable factorial structures from the EFA analyses, simple structure confirmatory factor analyses (CFA) were specified to investigate the external validity and distinctiveness of the MD factors. This was done by identifying the salient factor loadings for each of the factors from each of the selected EFA solutions and only allowing these symptoms to load on their respective factors in a CFA model while simultaneously regressing the factor(s) onto the 10 external covariates (Fig. 1). The CFA models were refit 10 times using different random seed values for the FIML Monte Carlo integration estimation procedure that was used. All analyses were performed in MPlus version 8.2 (Muthén and Muthén, 2017). A path diagram (Fig. S1) and further details are presented in the Supplemental Methods.

## 3. Results

### 3.1. Sample description

Of the 43,431 sample participants who completed the online survey, 60% were women, and their average age was 53.1 years (SD 12.8, Table 1). 11,110 subjects (26%) reported a two-week or longer period endorsing either or both depressed mood and/or interest loss at some point in their lives. About 90% or more of these 11,110 participants reported also having sleep problems, fatigue, or concentration difficulties in the period that they had depressed mood or interest loss (Table S3). Other symptoms that were often reported during that period of depressed mood or interest loss were non-DSM symptoms such as hopelessness, helplessness, libido loss, and feelings of sadness different from usual (>75%). A two-week period of experiencing only depressed mood or interest loss was very rare (*N* = 19, 0.001%). In total, 23% satisfied the criteria for an episode of MD according to the DSM-criteria during this two-week period, and 13% had sought professional help at some point during their lifetime.

### 3.2. Structure of 9 aggregated DSM symptom criteria for MD

The EFA of the 9 aggregated DSM symptoms indicated that a one factor solution fitted the data well, with all symptoms having substantial factor loadings (fl > 0.6) (Table S6). When a second factor was extracted, the 9 symptoms pulled apart into a ‘cognitive affective’ factor including depressed mood, interest loss, guilt and suicidality, and a ‘somatic’ factor including fatigue, concentration problems, appetite/weight problems, sleep problems, and psychomotor problems (Table S5a). These two factors were highly correlated (0.7), suggesting greater validity for the one factor solution.

**Fig. 2.**
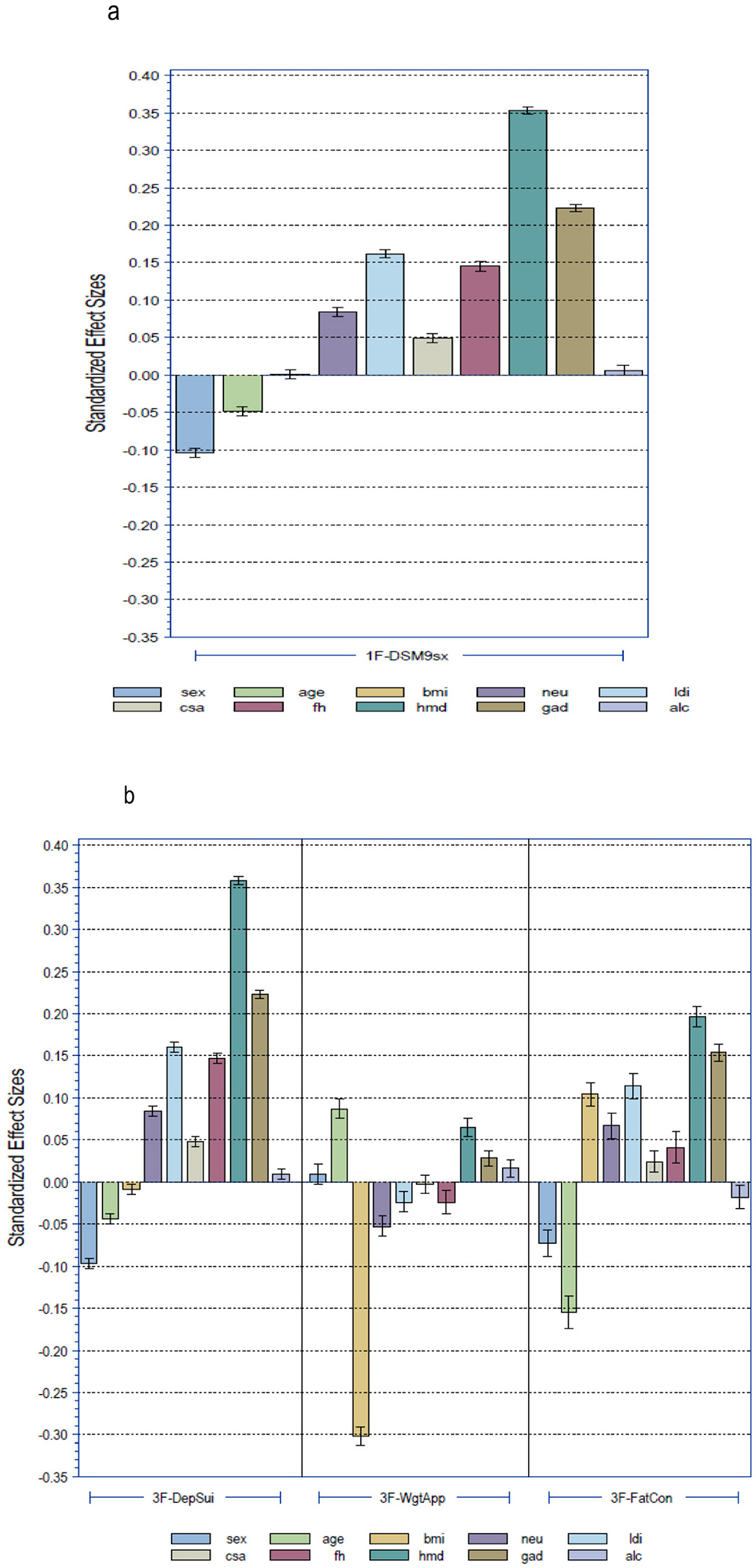

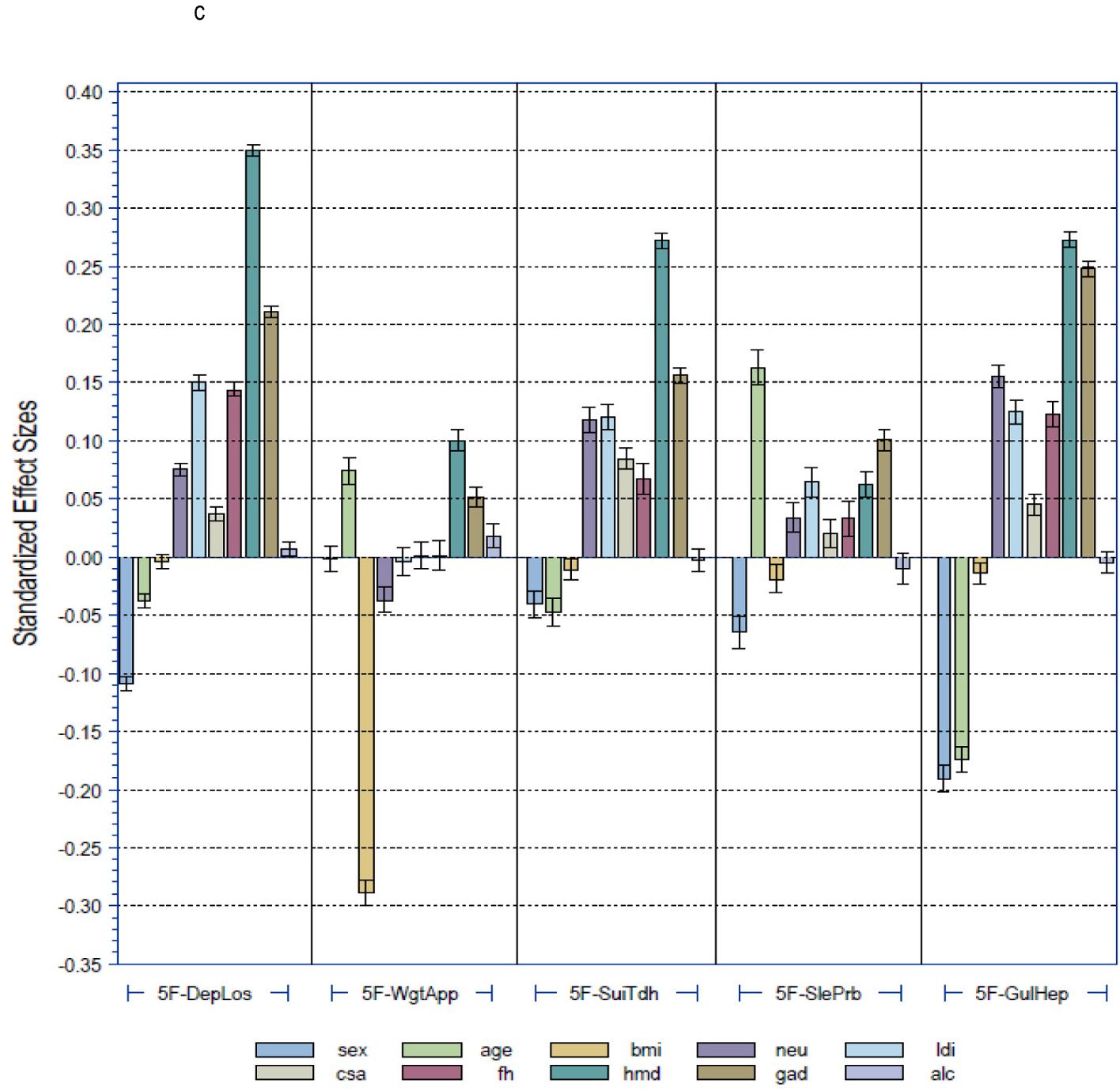
Graphic summary of CFA model standardized partial regression effect sizes for 10 risk external validators predicting the depressive symptom factors. a. Single factor model for 9 DSM depressive symptoms. b. Three factor model for 14 DSM depressive symptoms. c. Five factor model for 14 DSM & 9 non-DSM depressive symptoms.

Next, we investigated the association of this single factor with the external covariates using a CFA single structure analysis. This showed that a history of MD, a history of GAD, stressful life events and a family history of depression and/or anxiety were most strongly associated with this single dimension underlying the 9 aggregated DSM-A criteria (Fig. 2a). As expected, male sex and older age at assessment were negatively associated with this symptom dimension, similar to previous findings in a larger subsample of Lifelines (van Loo et al., 2021).

### 3.3. Structure of 14 disaggregated DSM symptom criteria for MDD

When we analyzed the 14 disaggregated DSM items in an EFA, all symptoms loaded moderately or strongly on a single factor (fl > |0.4|), except for hypersomnia (fl = 0.1) (Table S5b). Appetite and weight gain loaded negatively on this factor. When two or more factors were extracted, appetite and weight problems formed a separate dimension, with relatively low correlations with the other factors.

A three factor solution provided a good balance between statistical support and interpretability, as solutions of four or five factors led to separate factors with two items only indicating overextraction. The first dimension of this three factor solution included cognitive symptoms of depression (depressed mood, interest loss, guilt, suicidality) combined with insomnia and agitation (“depressed mood/suicidality”) (Table 2). The second dimension was marked by appetite/weight symptoms. The third dimension was defined by fatigue, concentration difficulties, hypersomnia, and retardation (“fatigue/concentration problems”). Inter-factor correlations for the oblique CFA version of the three factor model were modest (*r* < 0.3).

In the CFA model, the associations of the external covariates with the “depressed mood/suicidality” dimension resembled those found for the single factor solution of the 9 DSM symptoms in terms of direction and size of the effects (Fig. 2b). However, the “appetite/weight problems” dimension showed a different pattern and was most strongly associated with BMI but only minimally with the other covariates. The “fatigue/concentration problems” dimension was less strongly associated with risk factors of MD than the “depressed mood/suicidality” dimension, but it again was most strongly associated with a history of MD and GAD and also with younger age at interview.

### 3.4. Structure of 24 DSM and non-DSM symptoms

The EFA results fit to all 14 disaggregated DSM symptoms plus the 10 non-DSM symptoms showed moderate to strong loadings on a single factor (fl > 0.4), except for appetite gain, hypersomnia, and feeling worst in the morning (fl < 0.3, Table S5c). This last item was dropped from further analysis in the CFA due to its consistently poor loadings across all of the extracted factors. When two or more factors were extracted in the EFA, the appetite and weight symptoms again formed a separate dimension, with relatively low correlations with the other factors (*r* < 0.2). When three or more factors were allowed, most factors included a combination of DSM and non-DSM symptoms. Similar to the EFA solutions with 9 and 14 symptoms, the two core symptoms of depressed mood and anhedonia remained firmly together with high loadings across all factor extraction solutions.

The five factor solution indicated three correlated cognitive affective symptom dimensions –“depressed mood/anhedonia”, “suicidality/hopelessness”, “guilt/helplessness”– and two somatic symptom dimensions –“appetite/weight problems” and “sleep problems” (Tables 2, S6). The labels assigned to each factor were chosen based on the symptoms with the most salient factor loadings. All five dimensions included a mix of DSM and non-DSM symptoms, except for “appetite/weight problems” and “sleep problems”, which were exclusively marked by disaggregated DSM-symptoms. These somatic dimensions were not strongly associated with the cognitive affective dimensions (*r* ~ 0.1–0.3, CFA).

In a corresponding CFA model, the five factors were differentially associated with the ten external covariates (Fig. 2c). The “depressed mood/anhedonia” dimension was strongly associated with risk factors of MD, such as a history of MD and a history of GAD, stressful life events and a family history of depression and/or anxiety. This pattern of associations was similar to that observed in the single factor structure (9 symptoms) and to that of the “Dep/Sui” dimension of the three factor model (14 symptoms) (Fig. 2b). The two correlated cognitive affective depressive dimensions “suicidality/hopelessness” and “guilt/helplessness” were also quite strongly associated with a history of MD and a history of GAD. The somatic dimensions “appetite/weight problems” and “sleep problems” showed a distinct pattern. Both factors were not or only weakly associated with risk factors for MD, but strongly associated with BMI (appetite/weight problems) or age (sleep problems).

## 4. Discussion

### 4.1. Summary of main findings

We investigated the structure and clinical relevance of a broad set of depressive symptoms in a large general population sample during their worst lifetime depressive episode. We found evidence for a single factor underlying the 9 DSM symptoms for MD, but multiple factors were needed to adequately account for the correlations among the 14 disaggregated DSM symptoms and 24 DSM and non-DSM symptoms. In the analysis of the 24 depressive symptoms, three correlated cognitive affective symptom dimensions could be identified, which were most strongly associated with well-known risk factors for MD. The other two dimensions – appetite/weight problems and sleep problems — showed distinct patterns. These somatic dimensions were mainly associated with BMI and age, respectively, but were weakly associated with risk factors for MD.

### 4.2. Comparison with previous studies

Considering the type and number of depressive symptoms examined in this study, the most comparable study to date is a factor analysis of depressive symptoms in CONVERGE, a clinical sample of ~6000 Han Chinese women with recurrent major depressive disorder (Li et al., 2014). Despite differences in sample ascertainment (general population versus clinical population), sex (both sexes versus women only) and culture (Dutch versus Han Chinese), the extracted symptom dimensions were generally consistent. Factor loadings were comparable for the dimensions extracted from the 14 and 24 symptoms: 80% resp. 91% of the pairwise factor loading comparisons were similar (Table S7). One difference was that we found a single dimension adequately describing the 9 DSM-IV A-criteria of MD – comparable with previous studies in the general population (Aggen et al., 2005; Van Dam and Earleywine, 2011; Yu et al., 2012) — whereas in CONVERGE, two factors were extracted. However, the correlation between these two factors was high (0.78; CFA) which could be an argument for retaining a single factor instead of two factors. In sum, despite considerable differences across these two studies, the extracted dimensions and symptoms defining them were quite similar, indicating that the structure of depressive symptoms may be robust to differences in sex (Steen et al., 2021), cultural background, and severity of MD.

The degree of consistency when comparing our dimensional findings with CONVERGE was unexpected given the general lack of agreement in previous studies (van Loo et al., 2012). This structural congruence suggests that the inconsistency of factor structure findings in previous studies may be less due to the composition of the samples (clinical versus general population), and more to differences in other aspects of the study designs, such as relatively small sample sizes and the use of different questionnaires or structured interviews. To further test the robustness of the syndrome, replication studies should be conducted in both general population samples and clinical ascertained subjects with MD. Expanding the breadth of external validators to include to for instance age at onset or polygenic risk scores of MD also seems worthwhile.

### 4.3. Implications

First, our findings suggest that somatic symptoms of depression – specifically sleep, appetite and weight problems — are less specific indicators for MD than cognitive affective symptoms. The two somatic symptom dimensions describing appetite/weight and sleep problems were mainly associated with BMI and age, but only weakly linked with key risk factors of MD, in contrast to the cognitive affective symptom dimensions, which were mainly associated with risk factors for MD. This is in line with older descriptions of MD in the pre-DSM literature, in which less importance was attributed to sleep, appetite, and weight than in the DSM (Kendler, 2016). Although appetite loss, weight loss, and insomnia (but not appetite gain, weight gain or hypersomnia) were described as symptoms that may occur during MD, no authors noted them as key depressive symptoms in 19 psychiatric textbooks in the post-Kraepelin Western tradition (~1900–1960). Instead, these authors placed greater emphasis on cognitive and attitudinal changes such as worthlessness, hopelessness, and anxiety, which were all part of the cognitive affective dimensions and related to well-known risk factors of MD in our study.

The non-specificity of sleep, appetite and weight problems for risk of MD suggests that screening instruments for psychiatric conditions in the general population should place greater emphasis on the cognitive affective depressive symptoms compared to the appetite, weight or sleep problems for identifying subjects at risk for MD. These latter symptoms can be indicators of factors that appear to be distinctly different from those representing vulnerability for MD in a general population. For example, sleep problems in a general population may be reflective of aging (Roth, 2005), and may not necessarily act as an index of MD, although these problems are often observed in subjects with MD (Li et al., 2014; Steen et al., 2021). This suggests that instruments that rely more on somatic symptom dimensions to screen for MD in a general population sample may introduce more false positives than screening instruments that focus on the cognitive affective symptom dimensions. Furthermore, it suggests that simply counting depressive symptoms to form a single index of MD in a general population may not serve as a good proxy to identify individuals with MD (van Loo et al., 2021), which may also lead to biases when investigating the genetic architecture of MD (Cai et al., 2020). To better understand the etiology of the identified symptom dimensions, and assess their usefulness for defining MD (Kendler, 2013), future studies could focus on their possibly different genetic architectures, heritabilities, and correlations with other psychiatric and somatic traits and demographic or psychosocial factors.

Second, when we included both DSM and non-DSM symptoms in the factor analyses, all dimensions included one or more of the DSM-IV A criteria –similar to findings in CONVERGE (Li et al., 2014). This indicates that DSM items seem to capture the multidimensionality of MD relatively well. Note that this does not mean that DSM-items are sufficient to get an impression of the nature and severity of MD in clinical practice. The inter-item correlations between DSM- and non-DSM items are in general not very high, which means that the absence of certain DSM-items does not preclude the presence of non-DSM symptoms. Because non-DSM items may be indicators of a more severe prognosis in patients with MD (e.g., anxiety symptoms (First, 2011)), a thorough assessment of a wide range of symptoms in clinical practice is recommended.

### 4.4. Strengths and limitations

The strengths of this study include its large sample size from the general population, the broad set of depressive symptoms included in the analysis, and the CFA validation step in which we investigated the clinical relevance of the identified dimensions with external covariates.

We also note a number of limitations. First, although Lifelines is broadly representative of the general population of the north of the Netherlands (Klijs et al., 2015), respondents of our online survey among Lifelines participants tended to more often be female, were older and had higher educational attainment and income than non-respondents, which may weaken the representativeness of our study.

Second, we have not excluded participants with bipolar disorder, because diagnoses based on the DSM-criteria were not available in Lifelines. However, the potential impact of participants with bipolar disorder on the factor analysis results is likely to be minimal given the low prevalence of bipolar disorder of ~1.3% in the Dutch general population (de Graaf et al., 2012), and because our results were similar to those reported in CONVERGE in which subjects with major psychiatric disorders were excluded (Li et al., 2014).

Third, we dealt with missing data on depressive symptoms by using all available data of participants in a full-information maximum likelihood estimator with robust standard errors and Monte Carlo integration in an attempt to retain statistical inference and generalizability at the broader population level. Although missing data inevitably introduces more uncertainty into the statistical modeling results, it is reassuring that the parameter estimates (loadings, thresholds, inter-factor correlations) were relatively stable across refitting the factor models using different random seed values, and that the results showed some consistency with those reported in the CONVERGE study, in which there was almost no missing data (CONVERGE consortium, 2015).

Fourth, because we used retrospective reports asking subjects to recall their worst lifetime period of depressive symptoms, these responses could be subject to recall biases, but such memory related effects would only be problematic if they occur differentially for the individual depressive symptoms. In addition, the lifetime presence of depressive symptoms was based on participants’ self-reported answers on an online CIDI based instrument, which should not be taken as equivalent to a clinician rating the presence or absence of depressive symptoms.

Fifth, the identified symptom dimensions should not be interpreted as “real” or definite “entity-like” structures (Haeffel et al., 2022), but as indications how depressive symptoms cluster together and relate to external covariates. Factor analyses are subject to statistical underdetermination, meaning that multiple models may describe the data equally well, which may lead to unstable factor structures (Johnson, 2016; Romeijn and Williamson, 2018). However, the similarities between the dimensions reported in this study with those found in CONVERGE add some confidence to the robustness of these findings.

Sixth, strong correlations between appetite and weight loss and gain may contribute to multicollinearity and possible violations of the unidimensionality assumption of local independence (Supplemental Methods) (Van Loo et al., 2018). Although complete local independence is unlikely to be met in this (or any) empirical application, we see no evidence that the appetite/weight items have adversely impacted the resulting factor structures. If there was little information for other symptom dimensions besides appetite/weight problems, a two factor model with an appetite/weight and ‘all other symptoms’ dimension may have provided an adequate fit to these data, but this was not the case in our study. Further, the different predictions of the dimensions by the external risk factors add support and credibility to their distinctiveness.

## 5. Conclusion

We found qualitatively distinct depressive symptom dimensions in a general population who reported their lifetime worst period of depressive symptoms. These described three correlated cognitive affective dimensions mostly associated with risk factors for MD, and two somatic dimensions, mainly associated with age and BMI, but not or only weakly with risk factors for MD. Our findings in a Dutch general population sample were similar to findings in a large clinical sample of Han-Chinese women with recurrent MD, which suggests that the syndrome of MD may be relatively robust across sex, culture, and severity of MD.

## Supplementary Material

Appendix A

## Figures and Tables

**Table 1:** Sample description.

Variable	Prop/mean	Std err/dev	Min	Max	Skewness	Kurtosis	N	N Miss

Demographics								
Sex	0.40	0.002	0.00	1.00	0.41	−1.84	43,431	0
Age	53.09	12.81	18.00	92.00	−0.27	−0.11	43,431	0
Psychiatric history								
Depressed mood 2 wks^a^	0.22	0.002	0.00	1.00	1.33	−0.22	43,422	9
Interest loss 2 wks^a^	0.21	0.002	0.00	1.00	1.43	0.04	43,366	65
Diagnosed MD history^a^	0.12	0.002	0.00	1.00	2.27	3.15	43,431	0
MD Sx diagnosis history^a^	0.23	0.002	0.00	1.00	1.31	−0.29	43,422	9
GAD Sx diagnosis history^a^	0.09	0.001	0.00	1.00	2.86	6.19	43,420	11
Seek MD treatment	0.13	0.002	0.00	1.00	2.27	3.15	43,431	0
Risk factors								
Body mass index	25.79	4.30	8.66	82.33	1.57	6.58	43,249	182
Neuroticism	89.06	6.80	56.00	122.00	0.11	0.29	38,054	5377
Long-term difficulties (LDI)	1.92	1.98	0.00	8.00	1.11	0.65	37,796	5635
Childhood sex abuse	0.06	0.001	0.00	1.00	3.53	10.49	32,341	11,090
Family history MD	0.59	0.002	0.00	1.00	−0.36	−1.87	43,425	6
Heavy drinking	0.11	0.002	0.00	1.00	2.48	4.14	39,788	3643

Proportions are given for binary variables and means for continuous variables. Standard errors are given for binary variables (shown to 3 decimal places) and standard deviations are provided for quantitative variables (2 decimal places).

a Depressed mood 2 wks: Lifetime history of a period of depressed mood lasting two weeks or more; Interest loss 2 wks: Lifetime history of a period of interest loss lasting two weeks or more; Diagnosed MD history is assessed with the question “Have you ever been diagnosed with depression by a medical doctor or other health professional?” Instead MD Sx diagnosis history and GAD Sx diagnosis history are based on DSM-IV-TR criteria. We selected the one-item “diagnosed MD history” as external covariate in the CFA, instead of MD based on the DSM-criteria, because we already included the DSM-criteria as items in the CFA. Both were assessed in the online survey (see Supplemental Methods for details).

**Table 2:** CFA factor loadings.

Item	CFA DSM 9 aggregated	CFA DSM 14 disaggregated			CFA DSM 14 + nDSM 9 symptoms				
	F1	F1	F2	F3	F1	F2	F3	F4	F5

DSM symptoms									
Depressed mood	0.952*	0.963*	—	—	0.951*	—	—	—	—
Loss of interest	0.958*	0.954*	—	—	0.964*	—	—	—	—
Appetite/weight prob.	0.527*	—	—	—	—	—	—	—	—
Appetite gain	—	—	−0.954*	—	—	−0.944*	—	—	—
Appetite loss	—	—	0.750*	—	—	0.770*	—	—	—
Weight gain	—	—	−0.919*	—	—	−0.922*	—	—	—
Weight loss	—	—	0.792*	—	—	0.802*	—	—	—
Sleep problems	0.698*	—	—	—	—	—	—	—	—
Insomnia	—	0.345*	—	—	—	—	—	0.962*	—
Hypersomnia	—	—	—	0.412*	—	—	—	−0.384*	—
Psychomotor prob.	0.531*	—	—	—	—	—	—	—	—
Psychomotor retard.	—	—	—	0.576*	0.505*	—	—	—	—
Psychomotor agit.	—	0.349*	—	—	—	—	—	—	0.341*
Fatigue	0.760*	—	—	0.761*	0.738*	—	—	—	—
Concentration prob.	0.814*	—	—	0.682*	0.818*	—	—	—	—
Feelings of guilt	0.714*	0.701*	—	—	—	—	—	—	0.748*
Suicidal ideation	0.679*	0.699*	—	—	—	—	0.958*	—	—
Non-DSM symptoms									
Thoughts of death	—	—	—	—	—	—	0.785*	—	—
Different sadness	—	—	—	—	—	—	—	—	0.484*
Libido loss	—	—	—	—	0.554*	—	—	—	—
Reduced enjoyment	—	—	—	—	0.670*	—	—	—	—
Irritability	—	—	—	—	—	—	—	—	0.317*
Hopelessness	—	—	—	—	—	—	0.687*	—	—
Crying	—	—	—	—	—	—	—	—	0.357*
Helplessness	—	—	—	—	—	—	—	—	0.787*
Nervousness	—	—	—	—	—	—	—	—	0.581*
	F1	F1	F2	F3	F1	F2	F3	F4	F5
F1	1.000	1.000			1.000				
F2	–	0.190*	1.000		0.329*	1.000			
F3	–	0.265*	0.032	1.000	0.407*	0.116*	1.000		
F4	–	–	–	–	0.163*	0.148*	0.145*	1.000	
F5	–	–	–	–	0.615*	0.156*	0.595*	0.259*	1.000

agit., agitation; CFA, confirmatory factor analysis; MD, major depression; Prob., problems; retard., retardation. For a description of the assessed items we refer to Table S2 (Supplemental Material).

CFA Factor loadings for DSM 9 aggregated (single factor), 14 disaggregated (3 factors), and 14 DSM + 9 non-DSM (5 factors) MDD symptom model solutions when factors are predicted by each of the 10 external validators. The inter-factor correlations are Pearson Product Moment correlations for the latent continuous liability variables, and are directly estimated in the full factor model.

* Factor loading estimate statistically significant (α = 0.05). These point estimates of the factor loadings will differ to some degree from their EFA counterpart estimates due to: (1) the more restrictive nature of the CFA specification (i.e., no cross loadings allowed), and (2) these CFAs are estimate simultaneously when regressing each of the factors onto the 10 external predictors.

## References

[R1] Aggen SH, Neale MC, Kendler KS, 2005. DSM criteria for major depression: evaluating symptom patterns using latent-trait item response models. Psychol. Med. 35, 475–487. 10.1017/S0033291704003563. 15856718

[R2] American Psychiatric Association, 2013. Diagnostic and Statistical Manual of Mental Disorders, Fifth Edition: DSM-5, 5th ed. American Psychiatric Publishing, Washington, DC.

[R3] Balsis S, Cully JA, 2008. Comparing depression diagnostic symptoms across younger and older adults. Aging Ment. Health 12, 800–806. 10.1080/13607860802428000. 19023732

[R4] Barendse MT, Oort FJ, Timmerman ME, 2015. Using exploratory factor analysis to determine the dimensionality of discrete responses. Struct. Equ. Model. 22, 87–101. 10.1080/10705511.2014.934850.

[R5] Bot M, Middeldorp CM, de Geus EJC, Lau HM, Sinke M, van Nieuwenhuizen B, Smit JH, Boomsma DI, Penninx BWJH, 2017. Validity of LIDAS (LIfetime depression assessment self-report): a self-report online assessment of lifetime major depressive disorder. Psychol. Med. 47, 279–289. 10.1017/S0033291716002312. 27702414

[R6] Cai N, Revez JA, Adams MJ, Andlauer TFM, Breen G, Byrne EM, Clarke T-K, Forstner AJ, Grabe HJ, Hamilton SP, Levinson DF, Lewis CM, Lewis G, Martin NG, Milaneschi Y, Mors O, Müller-Myhsok B, Penninx BWJH, Perlis RH, Pistis G, Potash JB, Preisig M, Shi J, Smoller JW, Streit F, Tiemeier H, Uher R, Van der Auwera S, Viktorin A, Weissman MM, Kendler KS, Flint J, 2020. Minimal phenotyping yields genome-wide association signals of low specificity for major depression. Nat. Genet. 52, 437–447. 10.1038/s41588-020-0594-5. 32231276 PMC7906795

[R7] CONVERGE Consortium, 2015. Sparse whole-genome sequencing identifies two loci for major depressive disorder. Nature 523, 588–591. 10.1038/nature14659 [doi]. 26176920 PMC4522619

[R8] de Graaf R, ten Have M, van Gool C, van Dorsselaer S, 2012. Prevalence of mental disorders and trends from 1996 to 2009. Results from the Netherlands mental health survey and incidence Study-2. Soc. Psychiatry Psychiatr. Epidemiol. 47, 203–213. 10.1007/s00127-010-0334- [doi]. 21197531

[R9] De Jonge P, Ormel J, Van Den Brink RHS, Van Melle JP, Spijkerman TA, Kuijper A, Van Veldhuisen DJ, Van Den Berg MP, Honig A, Crijns HJGM, Schene AH, 2006. Symptom dimensions of depression following myocardial infarction and their relationship with somatic health status and cardiovascular prognosis. Am. J. Psychiatry 163, 138–144. 10.1176/appi.ajp.163.1.138. 16390901

[R10] De Miranda Azevedo R, Roest AM, Hoen PW, De Jonge P, 2014. Cognitive/affective and somatic/affective symptoms of depression in patients with heart disease and their association with cardiovascular prognosis: a meta-analysis. Psychol. Med. 10.1017/S0033291714000063. 24467963

[R11] Fedko IO, Hottenga J-J, Helmer Q, Mbarek H, Huider F, Amin N, Beulens JW, Bremmer MA, Elders PJ, Galesloot TE, Kiemeney LA, van Loo HM, Picavet HSJ, Rutters F, van der Spek A, van de Wiel AM, van Duijn C, de Geus EJC, Feskens EJM, Hartman CA, Oldehinkel AJ, Smit JH, Verschuren WMM, Penninx BWJH, Boomsma DI, Bot M, 2020. Measurement and genetic architecture of lifetime depression in the Netherlands as assessed by LIDAS (Lifetime depression assessment self-report). Psychol. Med. 10.1017/S0033291720000100. PMC8223240 32102724

[R12] First MB, 2011. DSM-5 proposals for mood disorders: a cost-benefit analysis. Curr. Opin. Psychiatry 24, 1–9. 10.1097/YCO.0b013e328340b594 [doi]. 21042219

[R13] Flora DB, Curran PJ, 2004. An empirical evaluation of alternative methods of estimation for confirmatory factor analysis with ordinal data. Psychol. Methods 9, 466–491. 10.1037/1082-989X.9.4.466. 15598100 PMC3153362

[R14] Fried EI, Nesse RM, 2015. Depression is not a consistent syndrome: an investigation of unique symptom patterns in the STAR*D study. J. Affect. Disord. 172, 96–102. 10.1016/j.jad.2014.10.010. 25451401 PMC4397113

[R15] Fried EI, Epskamp S, Nesse RM, Tuerlinckx F, Borsboom D, 2016. What are “good” depression symptoms? Comparing the centrality of DSM and non-DSM symptoms of depression in a network analysis. J. Affect. Disord. 189, 314–320. 10.1016/j.jad.2015.09.005. 26458184

[R16] Givens GH, Hoeting JA, 2013. Simulation and Monte Carlo Integration. In: Givens GH, Hoeting JA (Eds.), Computational Statistics. John Wiley & Sons, Inc, pp. 151–199. 10.1002/9781118555552.ch6.

[R17] Haeffel GJ, Jeronimus BF, Kaiser BN, , 2022. Folk classification and factor rotations: whales, sharks, and the problems with the Hierarchical Taxonomy of Psychopathology (HiTOP). Clin. Psychol. Sci. 10 (2), 259–278. 10.1177/21677026211002500. 35425668 PMC9004619

[R18] Havinga PJ, Boschloo L, Bloemen AJ, Nauta MH, de Vries SO, Penninx BW, Schoevers RA, Hartman CA, 2017. Doomed for disorder? High incidence of mood and anxiety disorders in offspring of depressed and anxious patients. J. Clin. Psychiatry 781. 10.4088/JCP.15m09936. 27898206

[R19] Hegeman AJM, Kok RM, Van Der Mast RC, Giltay EJ, 2012. Phenomenology of depression in older compared with younger adults: meta-analysis. Br. J. Psychiatry. 10.1192/bjp.bp.111.095950. 22474233

[R20] Johnson K, 2016. Realism and uncertainty of unobservable common causes in factor analysis. Noûs 50, 329–355. 10.1111/nous.12075.

[R21] Kendler KS, 2013. A history of the DSM-5 scientific review committee. Psychol. Med. 43, 1793–1800. 10.1017/S0033291713001578. 23822994

[R22] Kendler KS, 2016. The phenomenology of major depression and the representativeness and nature of DSM criteria. Am. J. Psychiatry 173, 771–780. 10.1176/appi.ajp.2016.15121509 [doi]. 27138588

[R23] Kendler KS, 2017. The genealogy of major depression: symptoms and signs of melancholia from 1880 to 1900. Mol. Psychiatry 22, 1539–1553. 10.1038/mp.2017.148. 28785109

[R24] Kendler KS, Gardner CO, Prescott CA, 2002. Toward a comprehensive developmental model for major depression in women. Am. J. Psychiatry 159, 1133–1145. 12091191 10.1176/appi.ajp.159.7.1133

[R25] Kendler KS, Gardner CO, Prescott CA, 2006. Toward a comprehensive developmental model for major depression in men. Am. J. Psychiatry 163, 115–124, 163/1/115 [pii]. 16390898 10.1176/appi.ajp.163.1.115

[R26] Kendler KS, Aggen SH, Neale MC, 2013. Evidence for multiple genetic factors underlying DSM-IV criteria for major depression. JAMA Psychiatry 70, 599–607. 10.1001/jamapsychiatry.2013.751. 23740048 PMC3800168

[R27] Kendler KS, Aggen SH, Flint J, Borsboom D, Fried EI, 2018. The centrality of DSM and non-DSM depressive symptoms in Han chinese women with major depression. J. Affect. Disord. 227, 739–744. 10.1016/j.jad.2017.11.032. 29179144 PMC5815309

[R28] Klijs B, Scholtens S, Mandemakers JJ, Snieder H, Stolk RP, Smidt N, 2015. Representativeness of the LifeLines cohort study. PLoS One 10, e0137203. 10.1371/journal.pone.0137203 [doi]. 26333164 PMC4557968

[R29] Li Y, Aggen S, Shi S, Gao J, Li Y, Tao M, Zhang K, Wang X, Gao C, Yang L, Liu Y, Li K, Shi J, Wang G, Liu L, Zhang J, Du B, Jiang G, Shen J, Zhang Z, Liang W, Sun J, Hu J, Liu T, Wang X, Miao G, Meng H, Li Y, Hu C, Li Y, Huang G, Li G, Ha B, Deng H, Mei Q, Zhong H, Gao S, Sang H, Zhang Y, Fang X, Yu F, Yang D, Liu T, Chen Y, Hong X, Wu W, Chen G, Cai M, Song Y, Pan J, Dong J, Pan R, Zhang W, Shen Z, Liu Z, Gu D, Wang X, Liu X, Zhang Q, Flint J, Kendler KS, 2014. The structure of the symptoms of major depression: exploratory and confirmatory factor analysis in depressed Han Chinese women. Psychol. Med. 44, 1391–1401. 10.1017/S003329171300192X [doi]. 23920138 PMC3967839

[R30] MacFadyen HW, 1975. The classification of depressive disorders. J. Clin. Psychol. 31, 380–394. 10.1002/1097-4679(197507)31:3&lt;380::AID-JCLP2270310302&gt;3.0.CO;2-1. 1100677

[R31] Milaneschi Y, Lamers F, Peyrot WJ, Baune BT, Breen G, Dehghan A, Forstner AJ, Grabe HJ, Homuth G, Kan C, Lewis C, Mullins N, Nauck M, Pistis G, Preisig M, Rivera M, Rietschel M, Streit F, Strohmaier J, Teumer A, Van Der Auwera S, Wray NR, Boomsma DI, Penninx BWJH, Ripke S, Mattheisen M, Trzaskowski M, Byrne EM, Abdellaoui A, Adams MJ, Agerbo E, Air TM, Andlauer TFM, Bacanu SA, Bakvad-Hansen M, Beekman ATF, Bigdeli TB, Binder EB, Blackwood DHR, Bryois J, Buttenschon HN, Bybjerg-Grauholm J, Cai N, Castelao E, Christensen JH, Clarke TK, Coleman JRI, Colodro-Conde L, Couvy-Duchesne B, Craddock N, Crawford GE, Davies G, Deary IJ, Degenhardt F, Derks EM, Direk N, Dolan CV, Dunn EC, Eley TC, Escott-Price V, Kiadeh FFH, Finucane HK, Frank J, Gaspar HA, Gill M, Goes FS, Gordon SD, Grove J, Hall LS, Hansen CS, Hansen TF, Herms S, Hickie IB, Hoffmann P, Horn C, Hottenga JJ, Hougaard DM, Ising M, Jansen R, Jorgenson E, Knowles JA, Kohane IS, Kraft J, Kretzschmar WW, Krogh J, Kutalik Z, Li Y, Lind PA, MacIntyre DJ, MacKinnon DF, Maier RM, Maier W, Marchini J, Mbarek H, McGrath P, McGuffin P, Medland SE, Mehta D, Middeldorp CM, Mihailov E, Milani L, Mondimore FM, Montgomery GW, Mostafavi S, Ng B, Nivard MG, Nyholt DR, O’Reilly PF, Oskarsson H, Owen MJ, Painter JN, Pedersen CB, Pedersen MG, Peterson RE, Pettersson E, Posthuma D, Quiroz JA, Qvist P, Rice JP, Riley BP, Mirza SS, Schoevers R, Schulte EC, Shen L, Shi J, Shyn SI, Sigurdsson E, Sinnamon GCB, Smit JH, Smith DJ, Stefansson H, Steinberg S, Tansey KE, Teismann H, Thompson W, Thomson PA, Thorgeirsson TE, Traylor M, Treutlein J, Trubetskoy V, Uitterlinden AG, Umbricht D, Van Hemert AM, Viktorin A, Visscher PM, Wang Y, Webb BT, Weinsheimer SM, Wellmann J, Willemsen G, Witt SH, Wu Y, Xi HS, Yang J, Zhang F, Arolt V, Berger K, Cichon S, Dannlowski U, De Geus EJC, DePaulo JR, Domenici E, Domschke K, Esko T, Hamilton SP, Hayward C, Heath AC, Kendler KS, Kloiber S, Lewis G, Li QS, Lucae S, Madden PAF, Magnusson PK, Martin NG, McIntosh AM, Metspalu A, Mors O, Mortensen PB, Muller-Myhsok B, Nordentoft M, Nothen MM, O’Donovan MC, Paciga SA, Pedersen NL, Perlis RH, Porteous DJ, Potash JB, Schaefer C, Schulze TG, Smoller JW, Stefansson K, Tiemeier H, Uher R, Volzke H, Weissman MM, Werge T, Levinson DF, Borglum AD, Sullivan PF, 2017. Genetic association of major depression with atypical features and obesity-related immunometabolic dysregulations. JAMA Psychiatry 74, 1214–1225. 10.1001/jamapsychiatry.2017.3016. 29049554 PMC6396812

[R32] Muthén LK, Muthén BO, 2017. Mplus User’s Guide, 8th ed. Muthén & Muthén, Los Angeles, CA.

[R33] Ohayon MM, Carskadon MA, Guilleminault C, Vitiello MV, 2004. Meta-analysis of quantitative sleep parameters from childhood to old age in healthy individuals: developing normative sleep values across the human lifespan. Sleep. 10.1093/sleep/27.7.1255. 15586779

[R34] Olbert CM, Gala GJ, Tupler LA, 2014. Quantifying heterogeneity attributable to polythetic diagnostic criteria: theoretical framework and empirical application. J. Abnorm. Psychol. 123, 452–462. 10.1037/a0036068 [doi]. 24886017

[R35] Roest AM, Thombs BD, Grace SL, Stewart DE, Abbey SE, de Jonge P, 2011. Somatic/affective symptoms, but not cognitive/affective symptoms, of depression after acute coronary syndrome are associated with 12-month all-cause mortality. J. Affect. Disord. 131, 158–163. 10.1016/j.jad.2010.11.018. 21159385

[R36] Romeijn JW, Williamson J, 2018. Intervention and identifiability in latent variable modelling. Minds Mach. 28, 243–264. 10.1007/s11023-018-9460-y. PMC6438491 30996521

[R37] Roth T, 2005. Prevalence, associated risks, and treatment patterns of insomnia. J. Clin. Psychiatry 66. 16336036

[R38] Scholtens S, Smidt N, Swertz MA, Bakker SJ, Dotinga A, Vonk JM, van Dijk F, van Zon SK, Wijmenga C, Wolffenbuttel BH, Stolk RP, 2015. Cohort profile: LifeLines, a three-generation cohort study and biobank. Int. J. Epidemiol. 44, 1172–1180. 10.1093/ije/dyu229. 25502107

[R39] Steen OD, van Borkulo CD, van Loo HM, 2021. Symptom networks in major depression do not diverge across sex, familial risk, and environmental risk. J. Affect. Disord. 10.1016/J.JAD.2021.07.002. 34303301

[R40] Van Dam NT, Earleywine M, 2011. Validation of the Center for Epidemiologic Studies Depression Scale-Revised (CESD-R): pragmatic depression assessment in the general population. Psychiatry Res. 186, 128–132. 10.1016/j.psychres.2010.08.018. 20843557

[R41] van Loo HM, de Jonge P, Romeijn JW, Kessler RC, Schoevers RA, 2012. Data-driven subtypes of major depressive disorder: a systematic review. BMC Med. 10, 156. 10.1186/1741-7015-10-156. 23210727 PMC3566979

[R42] van Loo HM, Van Borkulo CD, Peterson RE, Fried EI, Aggen SH, Borsboom D, Kendler KS, 2018. Robust symptom networks in recurrent major depression across different levels of genetic and environmental risk. J. Affect. Disord. 227, 313–322. 10.1016/j.jad.2017.10.038. 29132074 PMC5815316

[R43] Van Loo HM, Wanders RBK, Wardenaar KJ, Fried EI, 2018. Problems with latent class analysis to detect data-driven subtypes of depression. Mol. Psychiatry 23. 10.1038/mp.2016.202. 27821868

[R44] van Loo HM, Beijers L, Wieling M, De Jong TR, Schoevers RA, Kendler KS, 2021. Prevalence of internalizing disorders, symptoms, and traits across age using advanced nonlinear models. Psychol. Med. 10.1017/S0033291721001148. PMC9874996 33849670

[R45] Wanders RBK, Van Loo HM, Vermunt JK, Meijer RR, Hartman CA, Schoevers RA, Wardenaar KJ, De Jonge P, 2016. Casting wider nets for anxiety and depression: disability-driven cross-diagnostic subtypes in a large cohort. Psychol. Med. 46 10.1017/S0033291716002221. 27624913

[R46] Yu X, Tam WWS, Wong PTK, Lam TH, Stewart SM, 2012. The patient health Questionnaire-9 for measuring depressive symptoms among the general population in Hong Kong. Compr. Psychiatry 53, 95–102. 10.1016/j.comppsych.2010.11.002. 21193179

