# Supplementary material for: The structure of the symptoms of major depression: Factor analysis of a lifetime worst episode of depressive symptoms in a large general population sample
Source: J Affect Disord. Author manuscript; Available in PMC 2024 Feb 1. (PMC10833125; doi:10.1016/j.jad.2022.03.064)
Supplement: Appendix A [file NIHMS1929708-supplement-Appendix_A.pdf]

The structure of the symptoms of major depression:
factor analysis of a lifetime worst episode of depressive symptoms in a large general population sample
Hanna M van Loo, Steven H Aggen, Kenneth S Kendler

SUPPLEMENTAL MATERIAL

Table of Contents

SUPPLEMENTAL METHODS 2

Add-on study "Mood and mental health" (BIONIC2+) 2
External validators 2
Statistical Analysis 3
Exploratory factor analysis (EFA) 3
Figure S1: Path diagram illustrating EFA strategy 5
Missing data 6
Condition of local independence 7

SUPPLEMENTAL RESULTS 8

Table S1: Comparison between respondents and non-respondents BIONIC2+ 8
Table S2: Description of individual MD symptom items 9
Table S3: Descriptive summaries of depressive symptom items 10
Table S4: Summary of EFA Factor Extraction Model Fitting Results 12
Table S5a: EFA results 9 symptoms (1 to 3 Factors) 14
Table S5b: EFA results 14 symptoms (1 to 5 Factors) 15
Table S5c: EFA results 24 symptoms (1 to 6 Factors) 16
Table S6: EFA Factor loadings for 9 (1 factor), 14 (3 factors), 24 symptoms (5 factors) 17
Table S7: Comparison of Lifelines and CONVERGE EFA results 18

REFERENCES 21

Supplemental Methods

Add-on study "Mood and mental health" (BIONIC2+)

In the Summer of 2018, 124,330 adult Lifelines participants of whom a working email address was available were invited to complete an online questionnaire "Mood and mental health". This survey is also known as BIONIC2+, because it was an extension of the questionnaire used in the BIObanks Netherlands Internet Collaboration (BIONIC)(Bot *et al.* 2017; Fedko *et al.* 2020). After a reminder email, a total of 124,742 adult Lifelines participants were invited to participate. Of all invitees, 43,431 (34.8%) filled in the online questionnaire "Mood and mental health".

This online survey assessed the presence of lifetime MD, anxiety disorders, and other mental health related items. The survey consisted of a maximum of 119 questions. Questions were presented in a fixed order with pre-programmed skip patterns for items that were not applicable to reduce the time participants had to spend completing the survey and increase completion rate. Items had forced-choice response formats to reduce the number of missing values. Participants were offered the possibility to complete the survey in multiple stages, meaning that they could stop and continue at a later moment. For a comparison of characteristics between respondents and non-respondents, see Table S1. For a description of all individual depressive symptom items assessed in this survey and included in our study, see Tables S2 and S3.

External validators

Abb.	External covariates descriptions
SEX	Binary (0=Females; 1=Males)
AGE	Age at interview: range 18-92 years
NEU	Neuroticism sum score (depression and anxiety subscales NOT included)
LDI	Long-term difficulties inventory (sum score of 12 last year items)
CSA	Childhood sexual abuse (binary: (0=none, 1=low) = 0; (2=moderate, 3=severe) = 1)
FH	Family history (binary: 0 = no, 1 = at least one first degree family member history of depression and/or anxiety)
HMD	History of ever having an MD diagnosis (binary: 0=no, 1=yes)
GAD	History of generalized anxiety disorder diagnosis (binary: 0=no, 1=yes)
BMI	Body Mass Index
ALC	History of heavy drinking (binary: cutoff for males (≥ 2.5 drinks per day) & females (≥ 1.5 drinks per day))

Neuroticism (NEU) was assessed at baseline (between 2006-2013)(Scholtens *et al.* 2015) with the Revised Neuroticism-Extraversion-Openness Personality Inventory (NEO-PI) (Costa & McCrae 1992). For our neuroticism variable, we included all neuroticism subscales except the depression and

anxiety subscales, to prevent overlap with the depressive symptom items that were included in the EFA.

Long-term Difficulties Inventory (LDI) assessed 0-12 chronic stressors in the past year at baseline (Rosmalen *et al.* 2012).

Childhood sexual abuse (CSA) was assessed with the Childhood Trauma Questionnaire Short Form. (CTQ)(Bernstein *et al.* 2003). This measure was assessed with a separate questionnaire on average 5.6 years after baseline.

Family history (FH): Family history was assessed in the online survey "Mood and mental health" (2018) with items asking the participant "Did your [first degree relative] have problems with depression or anxiety? Yes/no (/I don't have any)" There were four separate items for biological father, biological mother, siblings, or children. If participants responded "yes" to at least one of the items, then this person was coded as having a family history with depression or anxiety.

History of MD (HMD) was assessed in the online survey "Mood and mental health" (2018) with the question "have you ever been diagnosed with depression by a medical doctor or other health professional?" For history of MD we selected the one-item self-reported history of MD as external covariate in the CFA, instead of MD based on the DSM-criteria, because we already included the DSM-criteria as items in the CFA.

History of GAD (GAD) was assessed in the online survey "Mood and mental health" (2018) according to DSM-IV-TR criteria. The items assessing lifetime symptoms of GAD were based on the MINI items used in previous Lifelines waves but were adapted to assess these symptoms lifetime instead of current.

Body Mass Index (BMI) was based on self-reported height and weight reported in the online survey "Mood and mental health" (2018).

History of heavy alcohol use (ALC): Alcohol intake at baseline (2006-2013) was assessed by questions about alcohol intake frequency and the average number of alcoholic drinks consumed on a drinking day.

Statistical Analysis

Exploratory factor analysis (EFA)

The primary aim of fitting the different EFA models was to characterize and obtain a more detailed understanding of the dimensional organization and break down of the DSM aggregated and disaggregate symptom criteria when analyzed within the context of additional non-DSM symptoms that can also characterize depression episodes. As displayed using path diagrams in Supplemental

Figure 1, the associations among the individual symptoms (square boxes) are “accounted for” or “reproduced” by fitting models with latent variables (circles) that decompose the item (co)variation into that which can be attributed to common latent variables (item shared variation) and that which is specific to each symptom (unique reliable item variance plus a random error). If the patterning of covariation among the symptoms can be better reproduced by introducing additional latent variables, such expanded factor structure should decrease the discrepancies between the covariation implied by the model and the actual observed covariation among the symptom items as derived from the sample data. In the EFA approach, both inter-factor correlations and factor-item cross-loadings are permitted (after rotation) allowing for a more complex means of reproducing the observed item association matrix. In exploratory analyses such as these that only place a minimal number of model restrictions to satisfy necessary identification requirements, there is a cost/trade-off for such a parameterization. These solutions are highly data-driven with minimal *a priori* theoretical guidance imposed on the model structures. The decomposition into two conceptually different sets of latent variables (i.e., common and item specific factors) provide a framework for interpreting the resulting EFA MD symptom item structures. A large item factor loading on one factor with near-zero cross-loadings on other factors and a corresponding small specific latent variable indicates the item is a salient indicator of what the variation on the common factor represents. Such an item has strong discriminating power in distinguishing between individual differences on the common factor in reference to the item threshold location.

EFA solutions typically return complex factor loading patterns after rotation with all items having a non-zero estimated factor loadings (i.e., many cross-loadings). In contrast, simple structure confirmatory specifications impose stronger restrictions by not allowing factor cross-loadings and forcing this model-based information to be absorbed into the factor inter-correlations. However, such a restrictive confirmatory model provides a more parsimonious representation of how the items function as indicators of the different dimensions of MD liability reducing the model complexity present in EFA solutions. Although such restrictions can simplify the interpretation of the modeling results with how the MD items mark the different factors, it comes with an increase in model-data misfit (i.e., the model implied covariation is unable to reproduce the observe inter-item covariance as well as the more highly parameterized EFA solution).

Figure S1: Path diagram illustrating EFA strategy

Schematic path diagram illustrating the sequential exploratory factor analyses strategy used to investigate the factorial space of the DSM aggregated, disaggregated and ten non-DSM depressive symptoms that may characterize depressive episodes.

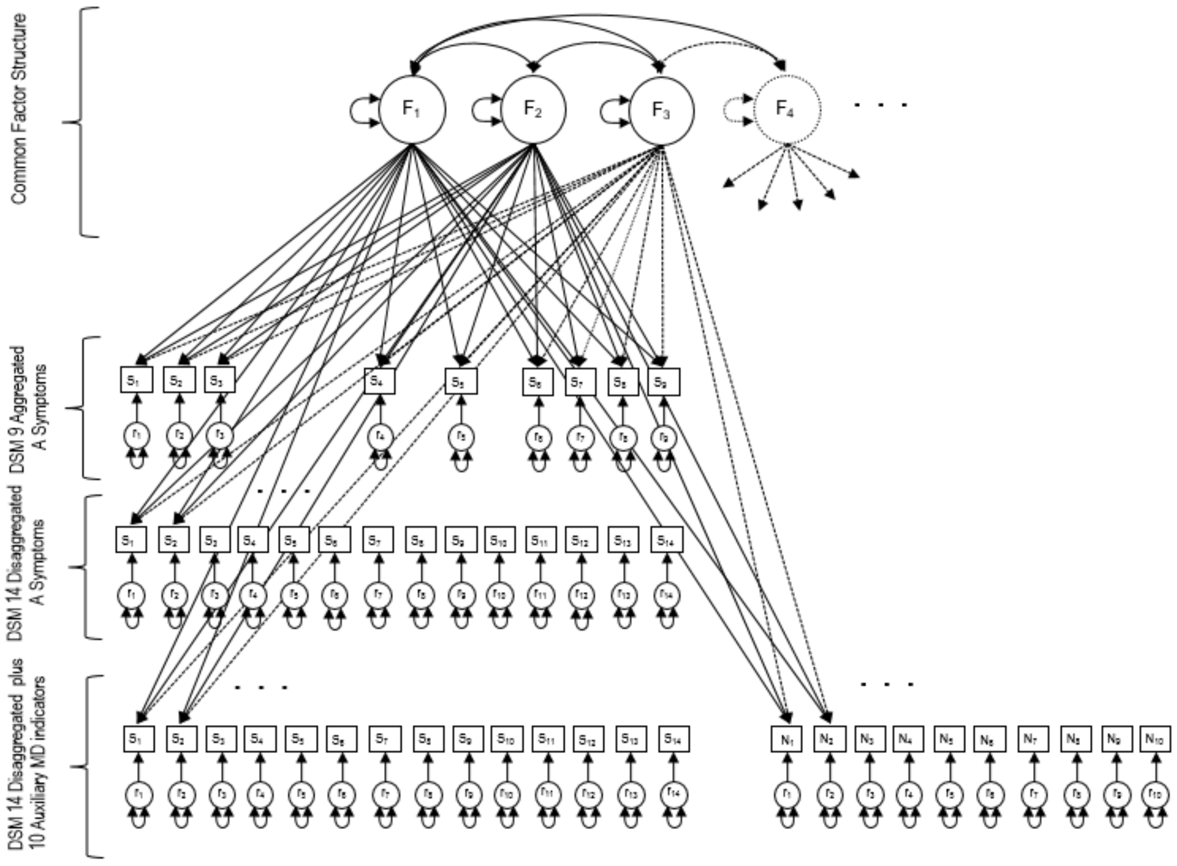

Square boxes = observed binary coded lifetime worst episode depressive symptoms ($S_{\#}$= DSM symptoms and $N_{\#}$ = non-DSM symptoms), Circles = latent variables ($F_{\#}$ = common factors, $r_{\#}$ = item specific factors), Single-headed arrows = regression-like factor loadings, Double-headed arrows = variances or covariances, Dashed arrows = indicates expansion when extracting additional factors, ". . ." = continuation of single-headed arrows (solid and dashed) going to the symptom items (squares) to reduce clutter in the diagram. Top left bracket = indicates the latent common factors, Lower 3 left brackets = indicates the 9 aggregated (symptoms labeled 1-9 first tier), 14 disaggregated DSM symptom sets (symptoms labeled 1-9 second tier), and the 10 non-DSM symptom items (symptoms labeled 10-19) set respectively that were submitted to exploratory factor analysis. Numbers in squares correspond to the symptom item descriptions provided in supplemental tables 1a and 2.

Missing data

The online web-based data collection protocol included a programmed conditional skip in/out feature to obtain lifetime present/absent responses to the MD symptom items. Only if participants positively endorsed either or both of the two core hierarchical criteria of depressed mood or anhedonia did they receive the remaining complement of items. This was done, first, to focus on additional depressive symptoms (like fatigue, concentration difficulties) that may co-occur in a period when subjects had at least one core symptom of MD. The assessment of lifetime periods of additional depressive symptoms of for instance fatigue, sleep problems, concentration problems may not always reflect a vulnerability for depression or mental disorders in general, and the prevalence may be extremely high (e.g. of fatigue) if people will be asked to report them during lifetime and not in combination with any of the core depressive symptoms. These symptoms may be part of different conditions, such as somatic illnesses, changing life circumstances (e.g. having young children), or stressful life conditions (covid, grieve), and are therefore very nonspecific if they do not occur during a period of other mental health symptoms. Second, since this online data collection effort was primarily targeted at collecting information to determine whether individuals met the clinical diagnosis criteria for MD affected or unaffected, the use of such a skip in/out protocol is commonly implemented.

However, when the focus is to understand the structural organization among symptoms in the general population, the main question is how to handle such missingness without introducing modeling selection effects. Carrying out multiple imputations to replace missing item values is one possible option. However, if only the information on the secondary items that do have data are used for the imputation, selection effects can be introduced if the subsample that endorsed either or both of the hierarchical symptoms is different from those saying no to both. Including additional variables other than the MD relevant items with more complete data is possible, but since many of these variables are used as external risk factors to evaluate the MD structure, including them in the imputation procedure could impact the estimated effect sizes for the external risk factors. A final consideration was that although the two "stem" items of depressed mood and loss of interest were included in the EFA and CFA modeling and theoretically met the definition for "data missing at random" under a full information perspective, these two items had no variation in their response values (i.e., both were zero).

Given the broader inferential aims of the study and considering the different possible strategies outlined above for dealing with the missing data structure, it was decided to use an estimation strategy that could appropriately model both the binary item-level information and accommodate the large amount of missing data for the secondary and auxiliary symptom items. Currently, there is a wide range of estimation procedures that are available to the data analyst. However, these estimation techniques incur some tradeoffs depending on the objectives of a study. Although limited information estimation techniques such as weighted least squares and its variants have become a useful option when modeling binary and ordinal data, it is less efficient when it comes

to dealing with large amounts of missing data. Full information maximum likelihood (FIML) is preferable, but when combined with standard dimensional integration techniques to estimate larger numbers of factors (e.g., 4 or more), this approach becomes computationally infeasible.

To deal with this missing data structure, we used a robust FIML estimator implemented using a Monte Carlo integration approach for the EFA and CFA analyses. The estimation technique is firmly seated in Bayesian probability and random variable parameter sampling theory (Muthén & Asparouhov 2012). The estimator uses all the available item response information and random resampling techniques to obtain posterior parameter distribution from which point estimates, standard errors and confidence intervals can be obtained.

Condition of local independence

The disaggregated symptoms of appetite and weight loss and gain consistently loaded on a separate factor, which may result from the inherently high correlations between these items (absolute correlations ranging from 0.4-0.9) whereas their correlations with the other symptoms was typically much smaller (mean correlation of ~0.2). To achieve local independence (i.e., independence among the symptoms given the latent factors), during EFA the appetite/weight items are pulled off onto their own factor in an attempt to minimize discrepancies between the observed and implied association matrices. Local independence is a theoretical principle often applied in factor and latent class models for evaluating unidimensionality. Technically, it states that after accounting for variation on the latent variable(s) scale(s), the individual items should be statistically independent. A practical and simple way to examine this principle is to examine the residual correlations summarizing the difference between the model-implied and observed correlations. In applications, it is rare that all residual correlations will be all exactly zero (McDonald & Mok 1995). Strong dependencies (multicollinearity) between items can impact latent variable models and introduce biases into parameter and posterior classification estimates (Van Loo *et al.* 2018). Although the condition of local independence is unlikely to have been completely satisfied in the analyses reported in this study, we do not think the disaggregated appetite/weight items and their correlational patterning have importantly impacted the factor structures found. If there was little information for other symptom dimensions besides appetite/weight problems, a two factor model with an appetite/weight and 'all other symptoms' dimension would have provided an adequate fit to these data, but this was not the case in our study. Further, the different patterns of associations of the dimensions with the external risk factors support the credibility of their distinctiveness.

SUPPLEMENTAL RESULTS

Table S1: Comparison between respondents and non-respondents BIONIC2+

	Responders	Non-responders	Statistic[1]	P-value[1]
Number	43,431	81,311		
Female sex (%)	60.0	57.4	74.8	<0.0001
Age (mean, SD)	53.7 (12.8)	48.0 (13.1)	-73.5	<0.0001
Age (range)	18 - 93	18 - 94		
Educational attainment (%)				
low	26.4	27.3	89.2	<0.0001
intermediate	39.2	41.8		
high	34.4	30.9		
Income (%)				
low	17.0	21.6	654.4	<0.0001
intermediate	55.3	57.2		
high	27.7	21.2		
MD or GAD at baseline (%)				
MD	1.62	2.12	35.6	<0.0001
GAD	3.57	4.39	45.9	<0.0001

Table S1 compares baseline characteristics of the respondents and the non-respondents of the online survey "Mood and mental health" (BIONIC2+, see Supplemental Methods p2). Age represents participants' age in 2018. Participants' age in 2018 was calculated by 2018 – year of birth. The responders' mean age in this table is thus slightly different than respondents' reported age in the BIONIC2+ survey and reported in Table 1.

Educational attainment represents the highest obtained degree as reported in wave 1. Low: no education, primary education, lower or preparatory secondary vocational education, junior general secondary education. Intermediate: secondary vocational education or work-based learning pathway, senior general secondary education, pre-university secondary education. High: higher vocational education, university education.

Income: The household equivalent monthly income was calculated by dividing the net household income by the square root of the number of persons living on this income. Low income: <1100 euro; intermediate: 1100-1899 euro; and high ≥1900 euro (Klijs et al., 2016).

Major depressive disorder (MD) and generalized anxiety disorder (GAD) were assessed with the MINI at wave 1 and represent the point prevalence of both disorders (MD in the past two weeks, GAD in the past six months).

Statistic and P-value were based on a chi-squared test for binary variables (sex, MDD, GAD), t-test for continuous variables (age), and a Cochran-Mantel-Haenszel test for ordinal variables (educational attainment, income).

Table S2: Description of individual MD symptom items

No.	Symptom label	Source	Symptom Description
1	Depressed mood	DSM	Depressed mood (almost every day, most of the day, for two weeks or more)
2	Loss of interest	DSM	Loss of interest (almost every day, most of the day, for two weeks or more)
3	Appetite/weight prob.	DSM	Appetite increase/decrease and/or weight gain/loss (any endorsed)
			All items below were assessed "During this period of two weeks or more of feeling depressed or having lost interest":
3a	Appetite gain	DSM	larger appetite than usual, almost every day
3b	Appetite loss	DSM	less appetite than usual, almost every day
3c	Weight gain	DSM	weight gain, at least one kilogram per week for several weeks
3d	Weight loss	DSM	loss of weight, at least one kilogram per week for several weeks, without trying
4	Sleep problems	DSM	Any sleep problems (insomnia and/or hypersomnia endorsed)
4a	Insomnia	DSM	Did you have trouble sleeping almost every night
4b	Hypersomnia	DSM	Were you sleeping too much almost every day
5	Psychomotor prob.	DSM	Any psychomotor problems (agitation and/or retardation endorsed)
5a	Psychomotor retard.	DSM	Did you talk or move more slowly than is normal for you
5b	Psychomotor agit.	DSM	Did you have to be moving all the time, i.e. couldn't sit still or pacing up and down
6	Fatigue	DSM	Did you lack energy or feel tired more than usual
7	Concentration prob.	DSM	Did you have a lot more trouble concentrating than usual
8	Feelings of guilt	DSM	Did you feel guilty or worthless
9	Suicidal ideation	DSM	Felt so low you thought about committing suicide
10	Thoughts of death	Other	Thought a lot about death, your own, someone else's, or death in general
11	Different sadness	Other	Feelings of sadness different from the usual feelings
12	Worst in morning	Other	Most days did you feel particularly bad when you first got up
13	Libido loss	Other	Was your interest in sex a lot less than usual
14	Reduced enjoyment	Other	Lose the ability to enjoy having good things happen to you
15	Irritability	Other	Did you feel irritable or angry most of the time
16	Hopelessness	Other	During that time did you feel hopeless about things
17	Crying	Other	During that time did you cry a lot
18	Helplessness	Other	During that time did you feel helpless much of the time
19	Nervousness	Other	During that time often felt nervous, jittery, or anxious

Note that "DSM symptoms" refer to the DSM A-criteria, and "non-DSM symptoms" refer to symptoms that are not part of the A-criteria.

Thoughts of death and suicidal ideation

The symptoms suicidal ideation and thoughts of death were both assessed in the BIONIC add-on study. Thoughts of death was assessed with the item "During this period of two weeks [in which you felt depressed or had a loss of interest], did you think often about death; about your own, or anyone else's death, or death in general?". Suicidal ideation was assessed with the item "During this period, did you often feel so bad that you thought about taking your own life?" We considered thoughts of death to be a symptom less specific for MD than suicidal ideation. Thoughts of death may for instance also occur if the subject (or the subject's relatives) have a high age or severe physical disorders, without being related to severe mood problems.

Table S3: Descriptive summaries of depressive symptom items

No.	Symptom	Count	Mean	StdErr	Skew	Kurtosis	N	Missing	Pct Miss
					DSM 9 Aggregated Symptoms				
1	Depressed mood	9664	0.223	0.002	1.33	-0.22	43422	0	0.000
2	Loss of interest	9083	0.209	0.002	1.43	0.04	43366	56	0.001
3	Appetite/weight prob.	6907	0.623	0.005	-0.51	-1.74	11078	32344	0.745
4	Sleep problems	9718	0.877	0.003	-2.29	3.25	11086	32336	0.745
5	Psychomotor prob.	4839	0.436	0.005	0.26	-1.94	11087	32335	0.745
6	Fatigue	10370	0.935	0.002	-3.53	10.48	11090	32332	0.745
7	Feelings of guilt	8154	0.736	0.004	-1.07	-0.85	11080	32342	0.745
8	Concentration prob.	10288	0.928	0.002	-3.31	8.97	11086	32336	0.745
9	Suicidal ideation	2914	0.263	0.004	1.08	-0.84	11083	32339	0.745
					DSM 14 Disaggregated Symptoms				
1	Depressed mood	—	—	—	—	—	—	—	—
2	Loss of interest	—	—	—	—	—	—	—	—
3a	Appetite gain	2263	0.205	0.004	1.46	0.15	11059	32363	0.745
3b	Appetite loss	4529	0.409	0.005	0.37	-1.86	11086	32336	0.745
3c	Weight gain	1244	0.112	0.003	2.46	4.04	11089	32333	0.745
3d	Weight loss	2260	0.204	0.004	1.47	0.16	11086	32336	0.745
4a	Insomnia	7872	0.710	0.004	-0.93	-1.14	11085	32337	0.745
4b	Hypersomnia	4143	0.374	0.005	0.52	-1.73	11080	32342	0.745
5a	Psychomotor retard.	3788	0.342	0.005	0.67	-1.56	11084	32338	0.745
5b	Psychomotor agit.	1764	0.159	0.003	1.86	1.47	11083	32339	0.745
6	Fatigue	—	—	—	—	—	—	—	—
7	Feelings of guilt	—	—	—	—	—	—	—	—
8	Concentration prob.	—	—	—	—	—	—	—	—
9	Suicidal ideation	—	—	—	—	—	—	—	—
					Non-DSM 10 MD Symptoms				
10	Thoughts of death	6153	0.555	0.005	-0.22	-1.95	11083	32339	0.745
11	Different sadness	8711	0.786	0.004	-1.40	-0.05	11076	32346	0.745
12	Worst in morning	3821	0.345	0.005	0.65	-1.58	11071	32351	0.745
13	Libido loss	8424	0.764	0.004	-1.25	-0.45	11019	32403	0.746
14	Reduced enjoyment	6906	0.624	0.005	-0.51	-1.74	11075	32347	0.745
15	Irritability	5457	0.493	0.005	0.03	-2.00	11079	32343	0.745
16	Hopelessness	8310	0.750	0.004	-1.16	-0.66	11078	32344	0.745
17	Crying	6377	0.575	0.005	-0.30	-1.91	11086	32336	0.745
18	Helplessness	8937	0.806	0.004	-1.55	0.40	11084	32338	0.745
19	Nervousness	7813	0.705	0.004	-0.90	-1.19	11082	32340	0.745

Sx Item = depressive symptom item, Mean = Symptom endorsement proportion, StdErr = Binary variable standard error, Skew = Item skewness, Kurtosis = item kurtosis N = number of observed responses, Missing = Number of missing values, Pct Miss = percentage of missing values

Table S3 provides a descriptive summary for each MD binary symptom item that was included in the sequence of EFA analyses carried out for participants with present data on this symptom. Note that for the additional depressive symptoms and the non-DSM symptoms we only had data from participants who endorsed having had a two-week period of depressed mood and/or interest loss (N=11,110).

All participants were asked whether they ever experienced a two week period/episode with depressed mood or interest less. But only participants who responded with affirmation were asked whether they had any other additional depressive symptoms during that same two week period. In total, 5% only endorsed depressed mood (N=2027), 3% only endorsed interest loss (N=1446), 18% endorsed both depressed mood and interest loss (N=7637), resulting in 26% participants who endorsed both depressed mood and/or interest loss (N=11,110).

For a description of the symptoms, see Table S2. “DSM symptoms” refer to the DSM A-criteria, and “non-DSM symptoms” refer to symptoms that are not part of the A-criteria.

Table S4: Summary of EFA Factor Extraction Model Fitting Results

Model	N par	Robust χ^2 Diff Test	df (p-value)	Log LL	AIC	BIC	sBIC	Sample Size
9 aggregated Lifelines DSM MD symptom items								
EFA1	18	—	—	–69768.4	139572.7	139728.9	139671.7	43422
EFA2	26	—	—	–69360.4	138772.8	138998.4	138915.8	43422
EFA3	33	—	—	–69292.2	138650.3	138936.7	138831.9	43422
1 vs. 2	—	857.3	8 (0.000)	—	–799.9	–730.5	–755.9	—
2 vs. 3	—	226.3	7 (0.000)	—	–122.4	–61. 7	–83.9	—
14 disaggregated Lifelines DSM MD symptom items								
EFA1	28	—	—	–99133.7	198323.4	198566.4	198477.5	43422
EFA2	41	—	—	–95289.6	190661.2	191017.0	190886.7	43422
EFA3	53	—	—	–94823.2	189752.3	190212.3	190043.9	43422
EFA4	64	—	—	–94146.2	188420.3	188975.8	188772.4	43422
EFA5	74	—	—	–94017.7	188183.5	188825.7	188590.5	43422
1 vs. 2	—	11815.9	13 (0.000)	—	–7662.2	–7549.4	–7590.7	—
2 vs. 3	—	402.7	12 (0.000)	—	–908.9	–804.7	–842.9	—
3 vs. 4	—	597.6	11 (0.000)	—	–1332.0	–1236.5	–1271.5	—
4 vs. 5	—	102.7	10 (0.000)	—	–236.9	–150.1	–181.7	—
24 (14 disaggregated DSM plus 10 non-DSM MD symptoms)								
EFA1	48	—	—	–162511.3	325118.7	325535.3	325382.7	43422
EFA2	71	—	—	–158431.9	317005.9	317622.0	317396.4	43422
EFA3	93	—	—	–157426.1	315038.2	315845.3	315549.7	43422
EFA4	114	—	—	–156074.1	312376.2	313365.6	313003.3	43422
EFA5	134	—	—	–155447.4	311162.8	312325.8	311899.9	43422
EFA6	153	—	—	–154820.8	309947.5	311275.4	310789.1	43422
1 vs. 2	—	7529.5	23 (0.000)	—	–8112.8	–7913.2	–7986.3	—
2 vs. 3	—	927.0	22 (0.000)	—	–1967.7	–1776. 8	–1846.7	—
3 vs. 4	—	1845.5	21 (0.000)	—	–2661.9	–2479.7	–2546.4	—
4 vs. 5	—	777.7	20 (0.000)	—	–1213. 4	–1039.8	–1103.4	—
5 vs. 6	—	1767.1	19 (0.000)	—	–1215.3	–1050.4	–1110.8	—

N par = number of estimated parameters (factor loadings & thresholds); Robust χ^2 Diff Test = adjusted robust chi-square difference test; df (p-value) = corresponding degrees of freedom and p-value for robust chi-square difference test; Log LL= raw data log likelihood; AIC = Akaike information criterion, BIC = Bayesian Information Criterion; sBIC = sample size adjusted BIC. All models were optimized using a robust full information maximum likelihood estimator implementing a Monte Carlo Integration approach to better deal with the large missing data that was generated by the interview skip-out protocol. Values presented are averaged across 10 different random seeds.

Table S4 provides a complete summary of the omnibus model fit indexes for evaluating the EFA common factor extraction modeling results for each of the 9, 14, and 24 MD symptom item indicator sets. The first column gives the number of factors extracted (e.g., EFA2 = 2 oblique factors) and different factor extraction comparisons (e.g., 1 vs. 2). What is evident in the omnibus summaries based on changes in the robust chi-square differences and information indexes AICs, BICs, and sample size adjusted BIC (sBIC), is that there is sufficient power to statistically justify extracting more factors in all 9, 14, and 24 symptom item sets. However, solutions with increasing numbers of factors were not always interpretable and better descriptions of the underlying structure than those with fewer factors in terms of parsimony, e.g., because such solutions included factors with only two item indicators, had high inter-factor correlations, or low factor loadings. Therefore, we selected EFA solutions that were a balance of acceptable statistical support and interpretability. This resulted in retaining a one factor solution for the 9 aggregated DSM symptoms, a three factor solution for the 14 disaggregated DSM symptoms and five factors for the 24 DSM and non-DSM symptoms.

Table S5a: EFA results 9 symptoms (1 to 3 Factors)

Item	f11	f12	f22	f13	f23	f33
Depressed mood	0.959*	0.998*	-0.013	0.937*	-0.007	0.056
Loss of interest	0.971*	0.863*	0.126*	0.893*	0.110*	-0.008
Appetite/weight prob.	0.636*	0.105*	0.487*	0.114	0.463*	0.037
Sleep prob.	0.757*	0.048	0.642*	0.047	0.628*	0.035
Psychomotor prob.	0.635*	-0.007	0.625*	-0.012	0.606*	0.047
Fatigue	0.815*	-0.163*	0.819*	-0.009	0.836*	-0.121
Concentration prob.	0.861*	0.061	0.743*	0.128	0.716*	-0.009
Feelings of guilt	0.715*	0.412*	0.334*	-0.001	0.184	0.629*
Suicidal ideation	0.696*	0.561*	0.202*	0.229	0.000	0.611*
EFA Inter Factor Correlations (Geomin oblique rotation)						
	F1	F1	F2	F1	F2	F3
F1	—	1.000		1.000		
F2	—	0.702*	1.000	0.714*	1.000	
F3	—	—	—	0.738*	0.654*	1.000

* = Averaged factor loading estimate statistically significant at $\alpha = 0.05$ level. Values highlighted in grey indicate the highest absolute factor loadings of that symptom across multiple factors. If the highest symptom factor loading is <|0.2| it is not highlighted, since such low factor loadings indicate that an item poorly discriminates individual differences on that factor. Due to the available power from the large sample size, even trivial non-discriminating factor loadings can be statistically significant. Column labeled headers beginning in column 2 indicate the factors extracted for each EFA (e.g., f11 = first (and only) factor for the single factor EFA, f12 = first factor for the 2 factor solution etc.). For a description of the symptom abbreviations, see Table S2.

Table S5b: EFA results 14 symptoms (1 to 5 Factors)

Item	f11	f12	f22	f13	f23	f33	f14	f24	f34	f44	f15	f25	f35	f45	f55
Depressed mood	0.959*	0.959*	-0.008	1.057*	0.009	-0.117*	1.041*	-0.021	-0.078	-0.014	0.949*	-0.020	0.001	-0.030	0.028
Loss of interest	0.970*	0.974*	-0.004	0.936*	0.045	0.006	0.924*	-0.036	0.045	0.013	0.948*	-0.004	0.049*	0.005	-0.013
Appetite gain	-0.502*	0.003	0.965*	-0.002	-0.916*	0.654*	0.005	0.974*	0.017	-0.023	-0.093	0.947*	0.007	-0.059*	0.097
Appetite loss	0.811*	0.758*	-0.622*	0.042	0.860*	0.149*	-0.004	-0.801*	0.718*	0.025	-0.010	-0.787*	0.730*	0.023	0.004
Weight gain	-0.420*	0.169*	0.890*	0.006	-0.817*	0.736*	0.014	0.887*	0.149*	-0.020	-0.006	0.891*	0.154*	-0.063	0.010
Weight loss	0.778*	0.706*	-0.673*	0.138	0.793*	0.005	0.013	-0.738*	0.628*	-0.101*	-0.023	-0.742*	0.612*	-0.120*	0.028
Insomnia	0.434*	0.429*	-0.057*	0.328*	0.096*	0.071	0.038	0.008	0.520*	-0.533*	0.052	0.008	0.382*	-0.624*	0.025
Hypersomnia	0.110*	0.261*	0.132*	-0.168	-0.051*	0.399*	0.120	-0.005	0.030	0.902*	0.007	-0.008	0.258*	0.807*	0.089
Psychomotor retard.	0.482*	0.605*	0.066*	-0.001	0.073*	0.578*	0.096	0.013	0.469*	0.136*	0.105	0.007	0.488*	0.108*	0.037
Psychomotor agit.	0.387*	0.428*	-0.036*	0.238*	0.091*	0.152*	0.096	-0.023	0.350*	-0.180*	0.005	-0.049*	0.289*	-0.207*	0.121
Fatigue	0.680*	0.780*	0.130*	-0.191	0.007	0.869*	-0.038	0.113*	0.645*	0.226*	0.065	0.129*	0.729*	0.150*	-0.127
Concentration prob.	0.806*	0.849*	0.040*	0.160	0.109*	0.619*	0.191*	0.000	0.605*	0.080	0.258*	-0.001	0.566*	0.060	0.019
Feelings of guilt	0.521*	0.675*	0.134*	0.520*	-0.074*	0.259*	0.510*	0.120*	0.211*	0.007	0.285*	0.062	0.136*	0.015	0.468*
Suicidal ideation	0.539*	0.660*	0.077*	0.656*	-0.039	0.107*	0.629*	0.053*	0.117*	-0.000	0.483*	-0.007	-0.023	0.010	0.512*

EFA Inter Factor Correlations (Geomin oblique rotation)

	F1	F1	F2	F1	F2	F3	F1	F2	F3	F4	F1	F2	F3	F4	F5
F1	—	1.000		1.000			1.000				1.000				
F2	—	0.108	1.000	0.397	1.000		0.031	1.000			-0.031	1.000			
F3	—	—	—	0.690*	0.280*	1.000	0.639*	0.245*	1.000		0.668*	0.232*	1.000		
F4	—	—	—	—	—	—	0.112	0.172	0.191	1.000	0.207*	0.085	0.266*	1.000	
F5	—	—	—	—	—	—	—	—	—	—	0.415*	0.156	0.416*	0.093	1.000

* = Averaged factor loading estimate statistically significant at $\alpha = 0.05$ level. Values highlighted in grey indicate the highest absolute factor loadings of that symptom across multiple factors. If the highest symptom factor loading is <|0.2| it is not highlighted, since such low factor loadings indicate that an item poorly discriminates individual differences on that factor. Column labeled headers beginning in column 2 indicate the factors extracted for each EFA (e.g., f11 = first (and only) factor for the single factor EFA, f12 = first factor for the 2 factor solution etc.).

Due to the large sample size power available, even trivial non-discriminating factor loading estimates can be statistically significant. Values in Red highlighted values indicate a Heywood case (negative residual variance. This condition is possible when using maximum likelihood estimation).

For a description of the symptom abbreviations, see Table S2.

Table S5c: EFA results 24 symptoms (1 to 6 Factors)

Item	f11	f12	f22	f13	f23	f33	f14	f24	f34	f44	f15	f25	f35	f45	f55	f16	f26	f36	f46	f56	f66
Depressed mood	0.955*	0.960*	-0.075*	1.041*	-0.012	-0.319*	0.761*	-0.049	0.272*	0.013	0.857*	-0.040*	0.180*	0.026	-0.037*	0.886*	-0.029*	0.015	0.069*	0.033*	0.052*
Loss of interest	0.953*	0.959*	-0.078*	1.066*	0.003	-0.348*	0.854*	-0.041	0.224*	-0.019	0.867*	-0.039*	0.181*	0.007	-0.014	0.882*	-0.021	0.071*	0.032	0.050*	0.030
Appetite gain	0.294*	0.002	0.960*	-0.014	0.895*	0.295*	0.052*	0.937*	0.020	0.177	0.205*	0.981*	0.022	0.054*	0.019	-0.065	0.949*	0.002	0.069*	0.057*	0.056*
Appetite loss	0.447*	0.703*	-0.626*	0.480*	-0.693*	-0.010*	0.268*	-0.708*	0.003	0.332*	0.097*	-0.767*	-0.005	0.043	0.480*	0.043	-0.769*	0.616*	0.044*	0.027*	0.010
Weight gain	0.409*	0.127*	0.899*	0.116	0.856*	0.264*	0.198*	0.872*	0.013	0.196*	0.391*	0.933*	-0.004	0.057*	0.029	0.076	0.908*	0.128*	0.087*	0.075*	-0.055
Weight loss	0.437*	0.655*	-0.663*	0.358*	-0.745*	0.064	0.125*	-0.748*	0.027	0.342*	0.034*	-0.754*	0.024	0.155*	0.394*	-0.010	-0.727*	0.481*	0.162*	0.073*	-0.015
Insomnia	0.476*	0.501*	-0.068*	0.044	-0.145*	0.348*	-0.183*	-0.114*	0.051*	0.427*	0.320*	0.016	0.022	0.626*	0.032	0.127	0.031*	0.002	0.689*	0.043*	0.060*
Hypersomnia	0.260*	0.224*	0.111*	0.469*	0.151*	-0.204*	0.474*	0.129*	0.036*	-0.051	0.003	-0.008	0.030*	-0.736*	0.484*	-0.028*	0.001	0.620*	-0.759*	0.035*	0.027*
Psychomotor retard.	0.571*	0.567*	0.030*	0.525*	0.028	0.015	0.403*	0.027*	0.051*	0.237*	0.333*	0.006	0.033	-0.086*	0.329*	0.181*	0.027*	0.489*	-0.067*	0.061*	-0.010
Psychomotor agit.	0.434*	0.452*	-0.041*	-0.003	-0.124*	0.400*	-0.213*	-0.088*	0.017	0.487*	-0.055	-0.037*	0.039	0.286*	0.292*	-0.207*	-0.033*	0.160*	0.291*	0.059*	0.201*
Fatigue	0.717*	0.694*	0.096*	0.663*	0.101*	0.009	0.577*	0.106*	-0.122*	0.370*	0.522*	0.093*	-0.159*	-0.093*	0.458*	0.229	0.143*	0.689*	-0.074*	-0.134*	0.014
Concentration prob.	0.828*	0.827*	-0.004	0.740*	-0.010	0.049	0.513*	-0.008	-0.011	0.422*	0.486*	-0.014	-0.027	0.003	0.453*	0.304*	0.008	0.475*	0.026	-0.061*	0.190*
Feelings of guilt	0.855*	0.833*	0.105*	0.600*	0.097*	0.269*	0.094	0.108*	0.293*	0.470*	0.015	0.100*	0.334*	-0.002	0.479*	0.062	0.087*	0.038	-0.007	0.125*	0.607*
Thoughts of death	0.777*	0.761*	0.010	0.665*	0.004	0.055	-0.044	-0.028*	0.865*	0.023	0.029	-0.018	0.837*	0.034	-0.011	-0.028*	-0.010	0.049*	0.040*	0.901*	-0.009
Suicidal ideation	0.848*	0.827*	0.058*	0.791*	0.057*	0.042	0.024	0.038*	0.982*	-0.002	0.038	0.022*	0.954*	-0.048*	0.029*	0.081*	0.020*	-0.009	-0.049*	0.852*	0.146*
Different sadness	0.672*	0.640*	0.077*	0.558*	0.082*	0.041	0.231*	0.077*	0.178*	0.240*	0.210*	0.069*	0.183*	-0.047*	0.251*	0.312*	0.063*	-0.013	-0.039*	0.006	0.369*
Worst in morning	0.139*	0.141*	0.002	-0.085	-0.036*	0.191*	-0.114*	-0.014	-0.075*	0.237*	-0.090	-0.005	-0.065*	0.071*	0.198*	-0.203*	-0.002	0.170*	0.076*	-0.020	0.061
Libido loss	0.587*	0.594*	-0.032*	0.535*	-0.033*	-0.004	0.383*	-0.038	0.032	0.249*	0.427*	-0.030*	0.012	0.043*	0.229*	0.314*	-0.009	0.320*	0.069*	0.008	0.018
Reduced enjoyoment	0.694*	0.703*	-0.062*	0.794*	-0.027	-0.171*	0.537*	-0.058*	0.231*	0.070	0.564*	-0.054*	0.197*	-0.005	0.077	0.574*	-0.041*	0.127*	0.014	0.108*	0.048*
Irritability	0.379*	0.332*	0.168*	0.037	0.121*	0.299*	-0.103*	0.150*	0.022	0.344*	-0.077	0.169*	0.046	0.106*	0.255*	-0.169*	0.165*	0.044	0.104*	0.001	0.300*
Hopelessness	0.906*	0.896*	0.037*	0.678*	0.016	0.267*	0.054	0.019*	0.441*	0.453*	-0.026	0.011	0.492*	0.034	0.456*	0.070	-0.006	-0.065*	0.018	0.226*	0.698*
Crying	0.463*	0.472*	-0.046*	0.285*	-0.071*	0.121*	0.083	-0.069*	0.066*	0.270*	0.012	-0.083*	0.078*	0.003	0.299*	0.015	-0.080*	0.122*	-0.000	0.008	0.280*
Helplessness	0.919*	0.914*	0.012	0.707*	-0.017	0.250*	0.180*	-0.010	0.258*	0.535*	0.030	-0.035	0.313*	-0.012	0.602*	0.130*	-0.049*	0.023	-0.032*	0.024	0.787*
Nervousness	0.690*	0.689*	0.003	0.197*	-0.079*	0.448*	-0.118*	-0.040	-0.010	0.671*	-0.018	-0.008	0.024	0.227*	0.509*	-0.118	-0.007	0.164*	0.225*	-0.054*	0.487*

EFA Inter Factor Correlations (Geomin oblique rotation)

	F1	F1	F2	F1	F2	F3	F1	F2	F3	F4	F1	F2	F3	F4	F5	F1	F2	F3	F4	F5	F6
F1	—	1.000		1.000			1.000				1.000					1.000					
F2	—	0.108*	1.000	-0.096	1.000		-0.082	1.000			-0.237*	1.000				-0.130*	1.000				
F3	—	—	—	0.409*	0.055	1.000	0.504*	-0.044	1.000		0.542*	-0.114	1.000			0.496*	0.189*	1.000			
F4	—	—	—	—	—	—	0.596*	0.129*	0.507*	1.000	0.044	-0.042	0.177	1.000		0.179*	-0.061*	0.349*	1.000		
F5	—	—	—	—	—	—	—	—	—	—	0.548*	0.099	0.440*	0.199*	1.000	0.522*	-0.025	0.345*	0.197*	1.000	
F6	—	—	—	—	—	—	—	—	—	—	—	—	—	—	—	0.636*	0.053	0.572*	0.241*	0.523*	1.000

* = Averaged factor loading estimate statistically significant at α = 0.05 level. Values highlighted in grey indicate the highest absolute factor loadings of that symptom across multiple factors. If the highest symptom factor loading is <|0.2| it is not highlighted, since such low factor loadings indicate that an item poorly discriminates individual differences on that factor. Column labeled headers beginning in column 2 indicate the factors extracted for each EFA (e.g., f11 = first (and only) factor for the single factor EFA, f12 = first factor for the 2 factor solution etc.). Due to the large sample size power available, even trivial non-discriminating factor loading estimates can be statistically significant. Values in Red highlighted values indicate a Heywood case (negative residual variance. This condition is possible when using maximum likelihood estimation). For a description of the symptom abbreviations, see Table S2.

Table S6: EFA Factor loadings for 9 (1 factor), 14 (3 factors), 24 symptoms (5 factors)

Item	EFA 9 Aggregated	EFA DSM 14 Disaggregated			EFA DSM 14 + 9 nDSM MDD Symptoms				
	F1	F1	F2	F3	F1	F2	F3	F4	F5
**DSM-symptoms**									
Depressed mood	0.959*	1.057*	0.009	-0.117*	0.857*	-0.040*	0.180*	0.026	-0.037*
Loss of interest	0.971*	0.936*	0.045	0.006	0.867*	-0.039*	0.181*	0.007	-0.014
Appetite/weight prob.	0.636*	——	——	——	——	——	——	——	——
Appetite gain	——	-0.002	-0.916*	0.654*	0.205*	0.981*	0.022	0.054*	0.019
Appetite loss	——	0.042	0.860*	0.149*	0.097*	-0.767*	-0.005	0.043	0.480*
Weight gain	——	0.006	-0.817*	0.736*	0.391*	0.933*	-0.004	0.057*	0.029
Weight loss	——	0.138	0.793*	0.005	0.034*	-0.754*	0.024	0.155*	0.394*
Sleep problems	0.757*	——	——	——	——	——	——	——	——
Insomnia	——	0.328*	0.096*	0.071	0.320*	0.016	0.022	0.626*	0.032
Hypersomnia	——	-0.168	-0.051*	0.399*	0.003	-0.008	0.030*	-0.736*	0.484*
Psychomotor prob.	0.635*	——	——	——	——	——	——	——	——
Psychomotor retard.	——	-0.001	0.073*	0.578*	0.333*	0.006	0.033	-0.086*	0.329*
Psychomotor agit.	——	0.238*	0.091*	0.152*	-0.055	-0.037*	0.039	0.286*	0.292*
Fatigue	0.815*	-0.191	0.007	0.869*	0.522*	0.093*	-0.159*	-0.093*	0.458*
Concentration prob.	0.861*	0.160	0.109*	0.619*	0.486*	-0.014	-0.027	0.003	0.453*
Feelings of guilt	0.715*	0.520*	-0.074*	0.259*	0.015	0.100*	0.334*	-0.002	0.479*
Suicidal ideation	0.696*	0.656*	-0.039	0.107*	0.038	0.022*	0.954*	-0.048*	0.029*
**Non-DSM symptoms**									
Thoughts of death	——	——	——	——	0.029	-0.018	0.837*	0.034	-0.011
Different sadness	——	——	——	——	0.210*	0.069*	0.183*	-0.047*	0.251*
Worst in morning	——	——	——	——	-0.090	-0.005	-0.065*	0.071*	0.198*
Libido loss	——	——	——	——	0.427*	-0.030*	0.012	0.043*	0.229*
Reduced enjoyment	——	——	——	——	0.564*	-0.054*	0.197*	-0.005	0.077
Irritability	——	——	——	——	-0.077	0.169*	0.046	0.106*	0.255*
Hopelessness	——	——	——	——	-0.026	0.011	0.492*	0.034	0.456*
Crying	——	——	——	——	0.012	-0.083*	0.078*	0.003	0.299*
Helplessness	——	——	——	——	0.030	-0.035	0.313*	-0.012	0.602*
Nervousness	——	——	——	——	-0.018	-0.008	0.024	0.227*	0.509*
	F1	F1	F2	F3	F1	F2	F3	F4	F5
F1	1.000	1.000			1.000				
F2	—	0.397	1.000		-0.237*	1.000			
F3	—	0.690*	0.280*	1.000	0.542*	-0.114	1.000		
F4	—	—	—	—	0.044	-0.042	0.177	1.000	
F5	—	—	—	—	0.548*	0.099	0.440*	0.199*	1.000

* = Average factor loading estimate statistically significant (α = 0.05). Due to the power that comes with very large sample sizes, even trivial factor loading estimates can be statistically significant from zero. The bottom rows present EFA inter-factor correlations (Geomin oblique rotation). Grey highlighted estimates indicate symptoms that have the highest loading on a factor. For a description of the symptom abbreviations, see Table S2.

Table S7: Comparison of Lifelines and CONVERGE EFA results

Table S7a: Comparison of Lifelines and CONVERGE EFA 2 factor solutions for 9 aggregated DSM symptoms

	Factor 1 Loadings		Factor 2 Loadings	
Item	LLF1	CVF1	LLF2	CVF2
Depressed mood	0.998	0.442	−0.013	0.363
Loss of interest	0.863	0.206	0.126	0.266
Appetite/weight prob.	0.105	0.002	0.487	0.511
Sleep prob.	0.048	−0.054	0.642	0.539
Psychomotor prob.	−0.007	0.020	0.625	0.647
Fatigue	−0.163	−0.210	0.819	0.719
Concentration prob.	0.061	0.070	0.743	0.661
Feelings of guilt	0.412	0.637	0.334	0.106
Suicidal ideation	0.561	0.657	0.202	-0.011
	F1	F2	F1	F2
F1	1.000		1.000	
F2	0.702	1.00	0.529	1.000

LLF1 = Lifelines Factor 1; CVF1 = CONVERGE Factor 1; LLF2 = Lifelines Factor 2; CVF2 = CONVERGE Factor 2 (Li *et al.* 2014)
Note that we found most evidence for a one factor solution to describe the 9 symptoms in Lifelines. But for a better comparison with the results in CONVERGE, we here present the results of the two factors that were extracted during EFA from the 9 symptoms in Lifelines.

Blue highlighted values indicate salient factor loadings that were deemed to be substantively relevant markers of the various depressive symptom factors. Factor loadings in red font indicate apparent discrepancies between the samples.

Table S7b: Comparison of Lifelines and CONVERGE EFA factor loadings for 3 factor solutions for 14 disaggregated DSM symptoms

	Factor 1 Loadings		Factor 2 Loadings		Factor 3 Loadings	
Item	LLF1	CVF1	LLF2	CVF2	LLF3	CVF3
Depressed mood	1.057	0.684	0.009	0.093	−0.117	−0.199
Loss of interest	0.936	0.408	0.045	−0.082	0.006	0.130
Appetite gain	−0.002	0.010	−0.916	0.738	0.654	0.366
Appetite loss	0.042	0.629	0.860	−0.722	0.149	0.000
Weight gain	0.006	−0.002	−0.817	0.842	0.736	0.338
Weight loss	0.138	0.398	0.793	−0.621	0.005	0.002
Insomnia	0.328	0.466	0.096	−0.003	0.071	−0.596
Hypersomnia	−0.168	−0.004	−0.051	0.016	0.399	0.765
Psychomotor retard.	−0.001	0.476	0.073	−0.105	0.578	0.099
Psychomotor agit.	0.238	0.458	0.091	−0.008	0.152	−0.134
Fatigue	−0.191	0.500	0.007	−0.100	0.869	−0.008
Concentration prob.	0.160	0.723	0.109	0.015	0.619	−0.055
Feelings of guilt	0.520	0.597	−0.074	0.048	0.259	0.086
Suicidal ideation	0.656	0.446	−0.039	−0.074	0.107	0.065
	LLF1	CVF1	LLF2	CVF2	LLF3	CVF3
F1	1.000	1.000				
F2	0.397	0.209	1.000	1.000		
F3	0.690	0.099	0.280	0.182	1.000	1.000

LLF1 = Lifelines Factor 1; CVF1 = CONVERGE Factor 1; LLF2 = Lifelines Factor 2; CVF2 = CONVERGE Factor 2, etc. (Li *et al.* 2014). Note that in our study we found evidence for a one factor solution describing the correlations among the 9 symptoms in Lifelines. For a more detailed comparison with the results in CONVERGE, we here present a comparison of the two factors for the EFA of the 9 symptoms in Lifelines with the two factor results from CONVERGE.

Blue highlighted values indicate salient factor loadings that were deemed to be substantively relevant markers of the various depressive symptom factors across the studies. Factor loadings in red font indicate apparent discrepancies between the samples. Values displayed in blue font are common cross-loadings (~ >0.3) across the 2 studies.

Out of 14 x 3 = 42 pairwise factor loading comparisons, 34 (80%) were similar, and 8 (20%) were deemed not to be similar.

Table S7c: Comparison of Lifelines and CONVERGE EFA factor loadings for 5 factor solutions for 14 disaggregated DSM symptoms plus (9 & 13) non-DSM symptoms

Item	LLF1	CVF1	LLF2	CVF2	LLF3	CVF3	LLF4	CVF4	LLF5	CVF5
Depressed mood	0.857	0.525	-0.040	−0.055	0.180	0.129	0.026	−0.187	-0.037	0.161
Loss of interest	0.867	0.437	-0.039	0.025	0.181	0.117	0.007	0.049	-0.014	−0.118
Appetite loss	0.097	0.398	-0.767	0.710	-0.005	−0.021	0.043	−0.016	0.480	−0.024
Weight loss	0.034	0.177	-0.754	0.647	0.024	−0.043	0.155	0.009	0.394	0.111
Appetite gain	0.205	−0.007	0.981	−0.653	0.022	0.002	0.054	0.560	0.019	0.188
Weight gain	0.391	0.093	0.933	−0.742	-0.004	−0.053	0.057	0.517	0.029	−0.020
Insomnia	0.320	0.396	0.016	0.061	0.022	−0.027	0.626	−0.489	0.032	0.143
Hypersomnia	0.003	−0.027	-0.008	−0.091	0.030	0.046	-0.736	0.575	0.484	−0.011
Psychomotor retard.	0.333	0.675	0.006	0.038	0.033	0.048	-0.086	0.085	0.329	−0.281
Psychomotor agit.	-0.055	0.334	-0.037	−0.033	0.039	0.012	0.286	−0.186	0.292	0.321
Fatigue	0.522	0.509	0.093	0.116	-0.159	−0.122	-0.093	0.041	0.458	0.044
C_Worthless	—	0.363	—	−0.016	—	0.435	—	0.113	—	0.110
Feelings of guilt	0.015	0.186	0.100	0.018	0.334	0.168	-0.002	0.163	0.479	0.258
Concentration prob.	0.486	0.721	-0.014	−0.056	-0.027	−0.105	0.003	−0.043	0.453	0.099
C_Slow thoughts	—	0.716	—	−0.180	—	0.016	—	−0.008	—	−0.028
C_Deciding	—	0.717	—	−0.099	—	0.031	—	−0.005	—	−0.004
Thoughts of deathl	0.029	−0.008	-0.018	−0.010	0.837	0.963	0.034	−0.072	-0.011	−0.025
Suicidal ideation	0.038	−0.011	0.022	−0.067	0.954	0.971	-0.048	−0.126	0.029	−0.010
Reduced enjoyment	0.564	0.409	-0.054	0.042	0.197	0.217	-0.005	−0.002	0.077	−0.140
Different sadness	0.210	0.139	0.069	0.103	0.183	0.112	-0.047	0.020	0.251	−0.122
Worst in morning	-0.090	0.217	-0.005	0.019	-0.065	−0.072	0.071	0.062	0.198	0.079
Libido loss	0.427	0.408	-0.030	0.020	0.012	0.042	0.043	−0.127	0.229	−0.044
Irritability	-0.077	−0.021	0.169	−0.065	0.046	0.011	0.106	−0.032	0.255	0.577
Hopelessnes	-0.026	0.211	0.011	0.068	0.492	0.572	0.034	0.086	0.456	0.199
Crying	0.012	−0.089	-0.083	0.140	0.078	0.168	0.003	0.143	0.299	0.368
Helplessness	0.030	0.199	-0.035	0.113	0.313	0.255	-0.012	0.190	0.602	0.386
Nervousness	-0.018	0.076	-0.008	−0.032	0.024	−0.002	0.227	−0.086	0.509	0.665

	LLF1	CVF1	LLF2	CVF2	LLF3	CVF3	LLF4	CVF4	LLF5	CVF5
F1	1.000	1.000								
F2	-0.237	0.063	1.000	1.000						
F3	0.542	0.378	-0.114	−0.081	1.000	1.000				
F4	0.044	0.130	-0.042	0.099	0.177	0.124	1.000	1.000		
F5	0.548	0.368	0.099	−0.001	0.440	0.223	0.199	0.020	1.000	1.000

LLF1 = Lifelines Factor 1; CVF1 = CONVERGE Factor 1; LLF2 = Lifelines Factor 2; CVF2 = CONVERGE Factor 2, etc. (Li *et al.* 2014)

Blue highlighted values indicate salient factor loadings that were deemed to be substantively relevant markers of the various depressive symptom factors. Factor loadings in red font indicate apparent discrepancies between the samples. Values displayed in blue font are common cross-loadings (~ >0.3) across the 2 studies.

Note that CONVERGE included 27 symptom items, whereas we included 24 symptom items in our EFA, because some DSM-items were assessed with disaggregated items in CONVERGE but were not assessed in Lifelines, such as C_worthless "worthlessness", C_Slow thoughts "slower/mixed-up thoughts", C_Deciding "Trouble making decisions".

Out of 24 x 5 = 120 pairwise factor loading comparisons, 109/120=91% were similar and 11 (20%) were not similar.

References

Bernstein DP, Stein JA, Newcomb MD, Walker E, Pogge D, Ahluvalia T, Stokes J, Handelsman L, Medrano M, Desmond D, Zule W (2003). Development and validation of a brief screening version of the Childhood Trauma Questionnaire. *Child Abuse and Neglect* **27**, 169–190.

Bot M, Middeldorp CM, de Geus EJC, Lau HM, Sinke M, van Nieuwenhuizen B, Smit JH, Boomsma DI, Penninx BWJH (2017). Validity of LIDAS (LIfetime Depression Assessment Self-report): a self-report online assessment of lifetime major depressive disorder. *Psychological Medicine* **47**, 279–289.

Costa PT, McCrae RR (1992). *Revised NEO personality inventory (NEO-PI-R) and NEO five-factor inventory (NEO-FFI). Odessa FL Psychological Assessment Resources*

Fedko IO, Hottenga J-J, Helmer Q, Mbarek H, Huider F, Amin N, Beulens JW, Bremmer MA, Elders PJ, Galesloot TE, Kiemeney LA, van Loo HM, Picavet HSJ, Rutters F, van der Spek A, van de Wiel AM, van Duijn C, de Geus EJC, Feskens EJM, Hartman CA, Oldehinkel AJ, Smit JH, Verschuren WMM, Penninx BWJH, Boomsma DI, Bot M (2020). Measurement and genetic architecture of lifetime depression in the Netherlands as assessed by LIDAS (Lifetime Depression Assessment Self-report). . Cambridge University Press *Psychological Medicine*

Li Y, Aggen S, Shi S, Gao J, Li Y, Tao M, Zhang K, Wang X, Gao C, Yang L, Liu Y, Li K, Shi J, Wang G, Liu L, Zhang J, Du B, Jiang G, Shen J, Zhang Z, Liang W, Sun J, Hu J, Liu T, Wang X, Miao G, Meng H, Li Y, Hu C, Li Y, Huang G, Li G, Ha B, Deng H, Mei Q, Zhong H, Gao S, Sang H, Zhang Y, Fang X, Yu F, Yang D, Liu T, Chen Y, Hong X, Wu W, Chen G, Cai M, Song Y, Pan J, Dong J, Pan R, Zhang W, Shen Z, Liu Z, Gu D, Wang X, Liu X, Zhang Q, Flint J, Kendler KS (2014). The structure of the symptoms of major depression: exploratory and confirmatory factor analysis in depressed Han Chinese women. . Wellcome Trust Centre for Human Genetics, Oxford, UK.; Virginia Institute for Psychiatric and Behavioral Genetics, Department of Psychiatry, Virginia Commonwealth University, Richmond, VA, USA.; Shanghai Mental Health Center, Shanghai, P.R. China (PRC).; *Psychological medicine* **44**, 1391–1401.

Van Loo HM, Wanders RBK, Wardenaar KJ, Fried EI (2018). Problems with latent class analysis to detect data-driven subtypes of depression. *Molecular Psychiatry* **23**

McDonald RP, Mok MM-C (1995). Goodness of Fit in Item Response Models. . Lawrence Erlbaum Associates, Inc. *Multivariate Behavioral Research* **30**, 23–40.

Muthén B, Asparouhov T (2012). Bayesian structural equation modeling: A more flexible representation of substantive theory. . Psychol Methods *Psychological Methods* **17**, 313–335.

Rosmalen JGM, Bos EH, de Jonge P (2012). Validation of the Long-term Difficulties Inventory (LDI) and the List of Threatening Experiences (LTE) as measures of stress in epidemiological population-based cohort studies. *Psychological medicine* **42**, 2599–608.

Scholtens S, Smidt N, Swertz MA, Bakker SJ, Dotinga A, Vonk JM, van Dijk F, van Zon SK, Wijmenga C, Wolffenbuttel BH, Stolk RP (2015). Cohort Profile: LifeLines, a three-generation cohort study and biobank. . ; all rights reserved. Published by Oxford University Press on behalf of the International Epidemiological Association: LifeLines Cohort Study, Groningen, The Netherlands, Department of Epidemiology, Genomics Coordination Center, Department of Genetics, Department of Internal Medicine, Department of Health Sciences, Community and Occupational Medicine and Department of End *International journal of epidemiology* **44**, 1172–1180.
